# Constructing concepts without feedback: An empirical investigation of how relational information affects multidimensional concept completion behavior in an unsupervised task

**DOI:** 10.1371/journal.pone.0328368

**Published:** 2025-08-07

**Authors:** Charles A. Doan, Ronaldo Vigo

**Affiliations:** 1 Department of Psychology, Marietta College, Marietta, Ohio, United States of America; 2 Department of Psychology, Ohio University, Athens, Ohio, United States of America; University of Bologna, ITALY

## Abstract

The ability of humans to intentionally learn, without feedback, unidimensional stimulus relations in categorization tasks has been empirically established over the past two decades. However, whether observers can learn more complex multidimensional stimulus relations across these unsupervised tasks has not yet been determined. We demonstrate across an unsupervised concept completion experiment that the failure to observe multidimensional learning in previous experiments may be attributable to factors such as increased stimulus or task complexity. We posit that concept completion is related to category learning in that it reveals the underlying tendencies that are associated with some categories being easier to learn than others. In our experiments, we found observers readily learned to complete a two-dimensional exclusive-or concept, evidenced by an increase in object selection as the task progressed with a decrease in choice response times. We also found that observers readily learned to complete, as evidenced by similar patterns in object selection and response time behavior, a more complex three-dimensional stimulus relation that has empirically been associated with large amounts of categorization errors in related supervised classification tasks. Accordingly, we tested two existing formal models to determine their ability to account for our observations: namely, the Simplicity Model and the Generalized Representational Information Theory (GRIT) basic measure. We show how relational information processing as expounded in GRIT accounts for the observed completion behavior. Overall, our findings show how people gravitate, in a gradual and composite fashion, towards minimizing the perceived complexity of categories as much as possible.

## Introduction

A near ubiquitous finding across unsupervised learning experiments is the ability for observers to increasingly isolate and utilize a single diagnostic dimension when categorizing multidimensional stimuli. More specifically, when observers are tasked with placing such stimuli into different groups without the aid of corrective feedback, they appear to utilize simple one-dimensional rules that are consistent with the underlying stimulus structure as specified a priori by researchers [1–4; but see 5]. Furthermore, observers display an increase in their ability to use such a simple rule, evidenced by lower proportions of classification errors as the category learning task progresses. The replicability of this one-dimensional sorting and learning result is not surprising given that this sorting is also the predominant type of behavior in related, but more unconstrained, unsupervised classification tasks focusing on sorting preferences [6–8]. What is surprising about this result, however, is that observers seem incapable of learning more complex types of relations without corrective feedback, such as relations involving two or more relevant stimulus dimensions [1,2]. Two important caveats to this broad characterization, however, are unsupervised categorization results from [9,10] who provide weak evidence that observers may be able to integrate the two relevant integral dimensions of brightness and saturation and two relevant auditory dimensions of duration and frequency formation, respectively. However, this two-dimensional behavior was rare among their participants and did not reach a level of high accuracy.

One of the main goals of the current work is to further assess the validity of the one-dimensional generalization by investigating whether multidimensional unsupervised learning is demonstratable when the task involves completing a partial multidimensional concept. This is important to consider because we often encounter new stimuli in our environment and must categorize these stimuli in relation to conceptual information that we have previously stored or processed, which is often multidimensional. Thus, rather than investigating whether observers can “build” or “create” concepts of multidimensional complexity from scratch, we are currently interested in whether observers can notice the multidimensional nature of a presented categorical stimulus and consistently modify it in a multidimensional manner.

As we discuss shortly, much of the research to date has utilized standard sequential learning procedures with stimulus sets more complex than theoretically necessary to observe two-dimensional and more complex unsupervised learning behavior. Instead, we take a different approach by utilizing simpler *Boolean* category structures (e.g., stimuli defined over binary-valued stimulus dimensions) with a novel sequential learning procedure that involves participants completing a partial concept presented to them. Consistent with prior research, we show that the tasks and category structures permit an investigation into unsupervised learning processes associated with completing one-dimensional and more complex two- and three-dimensional concepts [11,12]. Additionally, the current procedures assess learning these different types of concepts simultaneously – meaning that for any trial, an observer either implicitly or explicitly (e.g., via verbalization) chooses to complete the partial concept using a one-dimensional or multidimensional strategy. Our investigation is not the first to employ such a methodological strategy, as [2] constructed 2-dimensional stimulus sets whereby observers could either sort them by a one-dimensional or more complex two-dimensional strategy. Notwithstanding, we contend that the design of the current tasks coupled with the utilization of Boolean category structures represents a particularly promising empirical framework for assessing a wide range of potential unsupervised learning strategies, and not merely those explained in this report and assessed in previous research investigations.

A secondary goal of the current work is to provide a coherent theoretical explanation for the results from our experiments using formal models derived from *generalized invariance structure theory* (GIST; [13]) and *generalized representational information theory* (GRIT; Vigo, [13–16]). This general mathematical and computational theory of human conceptual behavior not only provides a naturally fitting framework with which to interpret the results from the current experiments but, as Vigo and associates [17–23] demonstrate, it provides reliable and tenable precedence in the way that its non-parametric and parametric formal models more accurately account for a wide range of learning results on two, three, and four-dimensional Boolean category structures than models derived from alternative theories. Additionally, the general notion of representational information conveyed in GRIT, and derived from GIST, has been successfully applied to account for and parsimoniously explain previous unsupervised sorting results across a myriad of experimental paradigms (to be discussed next) and for human informativeness judgments and information processing during categorization [18,21]. Thus, applying GIST and GRIT is a natural step forward for understanding unsupervised categorization and learning across the previous and current experiments. To be clear, we use GIST and GRIT because we know of no other theories that more naturally fit and account for our experimental results. For expositional clarity, we delay presenting the application of GRIT to the current experiment until the Discussion section.

### Selective review of unsupervised sorting and learning behavior

Before we describe in detail the current unsupervised learning experiment and the theoretical account of GIST and GRIT, we first summarize results from earlier and more recent unsupervised categorization tasks with similar stimuli and procedures. These procedures vary the constraints placed on the observer and whether the stimuli are presented sequentially or simultaneously. It is implied that each of these tasks is analogous to real-world situations that involve, in part, the same categorization and concept learning processes performed on encountered environmental (e.g., multidimensional) stimuli. At one extreme, the traditional unsupervised task involves observers either sorting an array of stimulus cards or a deck of stimulus cards into different groups in whatever way they choose and, thus, has zero constraints [6,24,25]. This task mimics situations where one encounters either multiple category instances (e.g., window shopping) or a singular category instance before making a decision regarding category membership. More generally, researchers regard the strategy adopted by participants in these tasks as preferences, rather than considering them learned responses [1]; however, there is a valid argument that even these preferences can be considered a learned response (discussed shortly). To clarify, by “strategy”, we simply mean the way in which the participants behavior can be categorized in a logical way. We are not implying that these strategies are completely verbalizable by observers for them to be able to use them.

A slightly constrained task involves instructing observers to place the stimuli into a set number of groups – usually two groups of equal size [1,7,8,26]. Regardless of this slight change, research shows observers use one stimulus dimension to categorize stimuli across both of these more relaxed unsupervised sorting procedures. However, as suggested by [27] and elaborated upon in [24], participants may be more likely to focus on one dimension that helps to contrast stimuli when they are asked to divide stimuli into two different categories, and this could result in the prevalent one-dimensional sorting behavior across these unsupervised categorization tasks. Consistent with this claim, [24,25] provide evidence of two-dimensional spontaneous categorization from an unsupervised classification task where they asked participants to divide the array of stimuli into any number of groupings that seemed natural to them (rather than a specific number such as 2). They found that the likelihood for participants to spontaneously categorize based on one or two dimensions depended upon the “intuitiveness” of each classification. More specifically, they found participants created one-dimensional classifications more often when those groupings were predicted by the simplicity model to be more intuitive compared to two-dimensional classifications, and that participants created two-dimensional classifications more often when those groupings were predicted by the simplicity model to be more intuitive compared to one-dimensional classifications.

These results show that one-dimensional or two-dimensional spontaneous classifications may appear depending on the abstract similarity structure of the stimuli and whether participants are constrained when dividing the set of stimuli into groups. One-dimensional sorting with these procedures where stimuli are presented and sorted simultaneous in an array, however, is generally robust to experimental manipulations designed to increase the prevalence of a different type of multi-dimensional sort, termed a “family resemblance” (FR) or “overall similarity” (OS) sort [7, 8; but see 26]. Indeed, the robustness of the one-dimensional result in these extreme situations has perplexed researchers, as evidence suggests our conceptual representations of natural kinds (e.g., animals or vehicles) may be organized in a manner analogous to the multidimensional FR structure [28–30]. More specifically, although this FR structure does not involve a dimension being perfectly diagnostic for classifying exemplars, it does involve most of the stimulus dimensions being relevant for classification; computationally, this structure maximizes similarity among exemplars within a category while minimizing similarity between exemplars of contrasting categories.

Although robust 1D sorting emerges with a simultaneous display and categorization of exemplars into two categories, modifying the procedure slightly can lead to FR sorting [8,31]. For this “match-to-standards” task, researchers display a prototypical stimulus for each of two categories and instruct participants to sequentially categorize exemplars beneath one of the two prototypes. The first instantiation of this task by Regehr and Brooks revealed robust FR-sorting when participants could not see how they partitioned their stimuli (placing exemplar cards face down), whereas robust 1D-sorting occurred when they could see their previous choices (situated in an array beneath each prototype). Subsequent research has revealed several factors that affect rates of FR-sorting with the match-to-standards task, including: variation in stimulus dimensions [31], time pressure [32], degree of participant impulsivity [33], and concurrent load [34].

Despite being important findings, the FR-sorting with the match-to-standards task and the robust 1D- and 2D-sorting observed in the tasks discussed above may be regarded as sorting preferences. Historically, this distinction is important because learning in unsupervised classification tasks has been traditionally operationalized as a relatively permanent change in participants response behavior with increasing experience with the presented category structures [1, 2]. However, the unsupervised preferences just discussed (1D, 2D, FR) reflect a more spontaneous and unchanging behavior from participants at the outset of the experiment. Despite this lack of change of preferences, one may validly argue participants bring with them into these unsupervised tasks a learned response/preference and that continuance of this preference across time is a demonstration of learned behavior (or the consistent application of an innate mechanism guiding our decisions). We agree with both characterizations of unsupervised learning, and we thank Emmanuel Pothos for bringing the importance of conveying this latter line of reasoning to our attention. We believe the current empirical framework is flexible to permit an analysis of both characterizations of unsupervised learning, though we primarily focus on the first characterization in this report.

Regarding the first characterization of learned unsupervised behavior, researchers have mirrored the presentation method of supervised tasks by presenting one stimulus at a time, rather than all of them simultaneously, and assessing how observers naturally “build” or “construct” categories [1,4,35–39]. For example, [1] instantiated four logical structures that were either separable by a one-dimensional rule or by a more complex two-dimensional relation. They found observers could learn each of these structures with the aid of corrective feedback, but that observers could only learn the one-dimensional structures when feedback was not provided. Although a consistent finding with many other unsupervised studies, they utilized continuous-valued stimulus dimensions (line length and orientation) and only assessed learning behavior for linearly separable categories. As we contend throughout the remainder of our report, employing these complex stimuli in conjunction with a small set of category structures may decrease the likelihood of finding different, and more complex, “rules” or strategies that observers can implement when feedback is not provided. To be clear, though these continuous-valued stimuli are perceptually simple to discern from each other, we suggest it may be more challenging for observers to discern multidimensional relationships between these stimuli when they can vary continuously rather than discretely.

One study supporting such an interpretation demonstrated unsupervised learning behavior more complex than the SD behavior just discussed [12]. They used a sequential presentation method for assessing both supervised and unsupervised learning; however, the logical structures were simpler and defined over three binary-valued stimulus dimensions. These structures, referred to as the SHJ structures for the first group of researchers to empirically assess their learning difficulty [39], involve six differentially complex and cognitively tractable learning problems. Each of these problems instantiate different types of learning behavior, such as categorization behavior consistent with SD, FR, and two-dimensional *exclusive-or* (henceforth, XOR) concepts. Across two different types of unsupervised learning tasks, Love found that recognition accuracy of category exemplars exceeded chance (50%) for each of these three category structures. Thus, observers were able to store aspects of the XOR and FR multidimensional relations when the exemplars were presented sequentially. Although an important and relevant finding for the current research, [12] assessed recognition accuracy rather than classification performance. Utilizing recognition accuracy does not permit an analysis of a change in behavior or strategy with increased experience with the category structure – the fundamental way of establishing unsupervised learning in categorization tasks to date. The current research extends upon the findings of [12] by reporting the results of a series of novel unsupervised categorization experiments that utilize the same Boolean structures as [12] and which have been incorporated in many other related categorization tasks [11,40,14,19,22,23,36–39,41–44].

### The current study

A primary goal of the current research is to assess whether unsupervised learning behavior more complex than 1D sorting emerges when the stimulus domain is simplified from objects consisting of continuous-valued dimensions to binary-valued dimensions and when the procedure is simplified to the minimal presentation of the categorical stimulus to observers. This minimal presentation is important because it ensures a maximum level of fidelity when processing the multidimensional relationships between stimuli, which should theoretically aid concept completion of multidimensional concepts.

We utilized a set of cognitively tractable *Boolean* category structures that instantiated several multidimensional sorting strategies more complex than 1D sorting (e.g., XOR, FR, and C-3D = complex three-dimensional) yet, arguably, less complex than the two-dimensional “diagonal” structure by [1] and other researchers. However, this is not to say that 1D-sorting strategies cannot be implemented with these more complex structures. 1D strategies are always possible to implement with Boolean structures, yet they are rarely deemed as optimal classification responses. Indeed, Ashby and colleagues suggest their participants may have been using such strategies with their more complex “diagonal” category structures and cite evidence from their experiments in support of the claim. Importantly, these structures vary in their levels of “logical coherence”, with a structure definable by a 1D sorting strategy containing the highest level of logical coherence [13,14,22]; discussed more fully in the Discussion]. The other structures, which are definable by a two-dimensional XOR, three-dimensional FR, and even more complex 3D relations, have lower levels of logical coherence and will be explicated at length in the Method section [11,23].

### Necessary design principles to elicit unsupervised learning?

[1] suggest there are four principles that must be met if experimental investigations into unsupervised learning are truly assessing *learning behavior* and not merely *preference behavior*, or the second characterization of unsupervised learning described above. Although the current experimental design differs in several important ways from those traditionally implemented by other researchers to date, we believe the current design satisfies the [1] constraints regarding proper investigations into the first characterization of unsupervised learning behavior.

**Fig 2:**
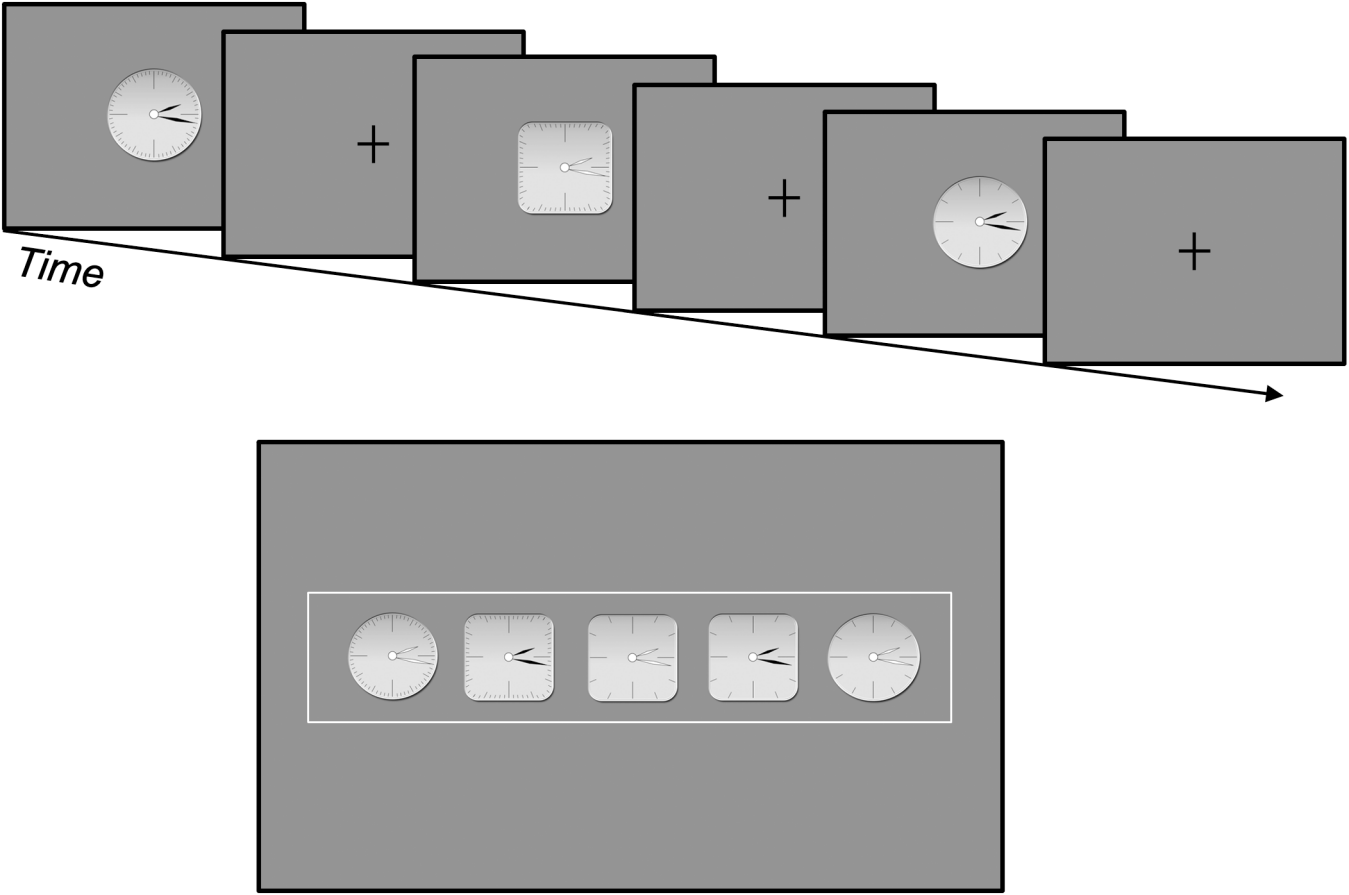
An example of completing the partial exclusive-or (XOR) concept.

The first principle is that “some underlying category structure must exist; that is, the exemplars of each contrasting category must form a coherent cluster in stimulus space” [1, p. 1178]. What conditions are necessary to ensure an “underlying category structure” exists and how can this be assessed a priori? Secondly, exactly what qualifies as a “coherent cluster in stimulus space” and are there objective measures that may delineate among different coherence levels? They implemented four distinct category structures – two whose membership was determinable along a single dimension and two requiring integrating information across two dimensions. It is visually clear from their plots of these structures [1, Fig 2, p. 1182] that each category specifies a cluster in multidimensional space and each cluster appears coherent.

Although the [1] categorical stimuli seem to satisfactorily address these questions and the first principle, there is an underutilized and more parsimonious method for operationalizing category structure. This method involves utilizing category structures whose stimulus dimensions are binary and whose structure adheres to principles underlying *Boolean* logic. These types of category structures have been employed extensively across varied experimental investigations of supervised human categorization behavior [20,39,42–44] and unsupervised categorization behavior [11,12,40,23,45–47]; see [48] for a detailed discussion]. We suggest their use in unsupervised learning experiments is promising for at least two reasons.

First, categorical stimuli consisting of objects defined over binary stimulus dimensions represent the simplest case of logical structure. Second, the logic underlying differences across *Boolean* category structures has been exhaustively specified up to categorical stimuli consisting of four stimulus dimensions [46,49]. This specification includes approximately 4 non-trivial two-dimensional structures, 20 non-trivial three-dimensional structures, and 400 non-trivial four-dimensional structures. With this wide range and exhaustive set of differentially complex and cognitively tractable category structures, it becomes readily apparent how the notion of “coherence” may be operationalized and systematically manipulated in experiments employing subsets of these structures. In addition, many of these Boolean category structures instantiate concepts that are not associated with clusters in multidimensional space (e.g., exclusive-or relation). This is an important distinction, as these structures provide a means to assess whether the “cluster” criterion explicated in the first Ashby et al. constraint is strictly necessary from a methodological standpoint when assessing the limits of unsupervised learning behavior in humans.

**Fig 1:**
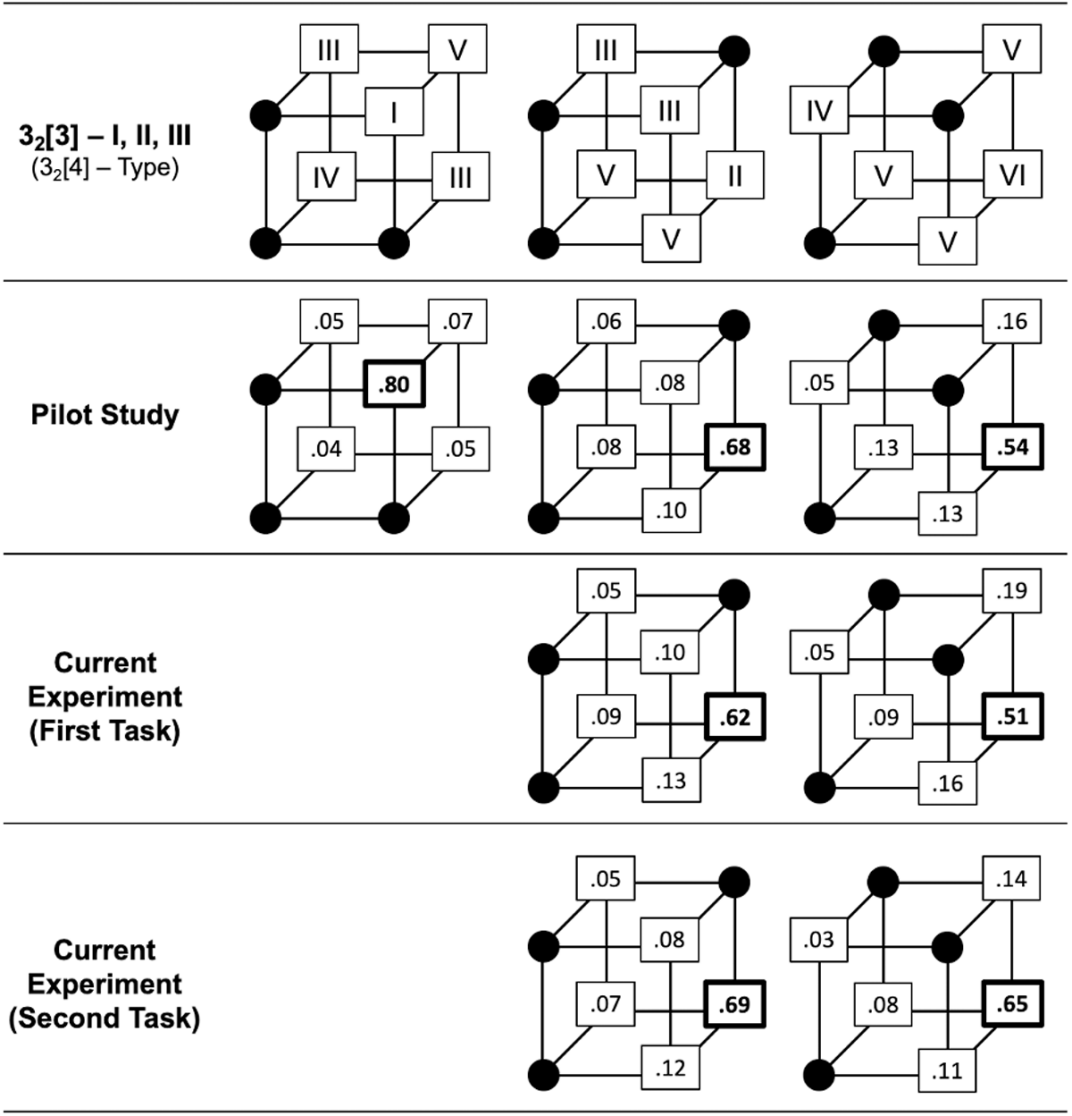
Boolean cube depiction of the partial one-dimensional (1D), exclusive-or (XOR), and complex three-dimensional (C-3D) concepts assessed in the current experiments.

As an example, Fig 1 displays Boolean cubes associated with three of these three-dimensional category structures, where each structure consists of three category members defined by binary-valued stimulus dimensions. The black dots on each cube represent these three members for each structure, whereas the corners without black dots (e.g., those with boxes containing roman numerals) represent objects not belonging to the category structure. Each axis of the cube (*x, y, z*) represents the two possible values for a particular stimulus dimension. We can use the general “*D*_*n*_[p] – Type” notation to represent each category structure, where *D* stands for the number of dimensions composing each object stimulus (3 for the current study), *n* stands for the number of possible values per dimension (2 for Boolean), and [p] stands for the number of positive examples of the presented category structure (3 for the current study; 14, 46). The roman numeral within the notation represents the distinct logical configuration (e.g., Type) associated with that grouping of category members. For the three category structures shown in Fig 1 and that were used in the current study, their category structure notation is 3_2_[3] – I, 3_2_[3] – II, and 3_2_[3] – III and these are associated with partial 1D, XOR, and C-3D concepts.

For the current study, each corner of each cube represents a three-dimensional object (e.g., a realistic clock/t-shirt across the current experiments). The black dots on each cube represent the objects belonging to the 3_2_[3] type (I, II, and III, respectively; partial concepts of 1D, XOR, C3D, respectively) that were presented sequentially during each experiment (row 2 = Pilot Study; rows 3/4 = Current Experiment). The squares with roman numerals (I-VI; row 1) represent the objects that were possible for participants to select, with each selection creating one of the six 3_2_[4] category structures when there are four black dots on the cube (I-VI; proportions given in rows 2–4). The bolded values and heavily bolded boxes in rows 2–4 represent the most-often chosen object, statistically, as revealed by chi-square goodness-of-fit tests (3_2_[4] – I is completing the 1D concept, 3_2_[4] – II is completing the XOR concept, and 3_2_[4] – VI is completing the C-3D concept). For reference, the number of possible decisions for each cube is *N* = 2,592 for row 2, *N* = 2,304 for the middle cube for rows 3 and 4, and *N* = 2,208 for the rightmost cube for rows 3 and 4.

Regarding coherence, the category structure associated with the leftmost cube (3_2_[3] – I) may be considered the most coherent (all objects share the property for the z^th^-dimension), while the category structures associated with the other two cubes may be considered less coherent. Furthermore, as one can readily see from the Boolean cubes in columns 2 and 3, this notion of “coherence” as applied to Boolean categories does not necessitate each category being separated by a linear boundary in multidimensional space. For example, the 3_2_[3] – II category structure (middle column) is not linearly-separable, regardless of which object is chosen to complete the concept (creating a 3_2_[4] category structure); whereas, type 3_2_[3] – III (rightmost column) is linearly-separable if participants choose the top-left corner object to complete the concept (creating type 3_2_[4] – IV). Foreshadowing the results of the current experiment, the proportions given in rows 3–4 show robust XOR concept completion by participants (creating type 3_2_[4] – II) and C-3D concept completion (creating type 3_2_[4] – VI) for these two category structures. This distinction is important when considering that the category structures utilized in most unsupervised tasks to date are separated by such a boundary and form separate clusters in multidimensional space; but the current data suggest that ensuring categorical stimuli occupy separate clusters in MD-space is not strictly necessary when investigating unsupervised learning behavior.

The second principle for studying constraints on unsupervised learning behavior is that “observers must be given extensive practice with the category structures” [1, p. 1178]. They addressed this principle by randomly presenting 80 stimuli (40 for each of two categories) during 5 odd-blocked experimental sessions. They instructed participants to simply observe the stimuli during these sessions and to “try to learn about the two categories” [1, p. 1184]. They surmised that any observable changes in classification behavior during even-blocked sessions would be the result of what participants progressively gleaned about the underlying category structure in the odd-blocked sessions (e.g., learning). Their approach in addressing this principle is certainly reasonable and their alternating-block method is one way of ensuring participants are given extensive practice with the underlying category structures. A related method involves merging the observational and classification blocks into one block and assessing whether the classification behavior changes as multiple instantiations of the structure are shown throughout the task. With this method, the entire underlying category structure is presented (observation) and participants make a decision regarding how to modify this structure (categorization). Learning can be assessed by determining whether the pattern of modification decisions changes as the task progresses; that is, whether modification decisions change as a function of increased exposure to the same underlying category structure during successive observations. Regarding the Boolean cube representations in Fig 1, this is analogous to adding a fourth black dot to each cube (e.g., creating the six logically different 3_2_[4] category structures) and assessing whether the creation of these new four-object logical configurations systematically changes as observers gain more experience with the stimuli and task. Currently, we report results across our experiments utilizing this type of experimental method and we refer to the task as completing the partial concepts (1D, XOR, C-3D) that are presented during the observation phase. We assessed the impact of “extensive practice” for completing each of these partial concepts by presenting a total of 48 trials for each of their underlying category structures. This method of inducing practice is novel to the current study and, as such, we operationalized learning similar to how [2] operationalized learning in their unsupervised sorting task – as an increase in similar object selection with increasing trials in the experiment. Importantly, however, we also recognize there are plausible alternative explanations for the behavior observed in the current tasks that do not instantiate these partial concepts, and which may involve little to no learning of interrelationships between stimulus dimensions. We discuss this limitation and one of these explanations at length in the Discussion section.

The third principle is that “the observer should be told there is an underlying category structure” [1, p. 1179]. They notified participants that they should look for how to partition the set of 80 stimuli into two equally-sized categories. This principle, however, seems incompatible with how unsupervised learning may occur in real-world contexts where an observer may not be explicitly told to look for relations between the exemplars they are encountering. Notwithstanding this objection, a reasonable counter is that observers are merely responding with their preferences in these real-world contexts, and these do not systematically change across time. But exactly how do these preferences come about in the first place? We suggest the more general experimental approach for assessing the presence of unsupervised learning be to withhold explication of this principle to participants. The fourth principle is inexorably linked with the third principle, stating that “the observer should be encouraged to respond as accurately as possible” [1, p. 1179]. Needless to say, if one is not told that there is an underlying category structure, then they should also not be told to respond accurately. Though we adopted this relaxed version of principles three and four in our pilot study by encouraging consistency in responding (described next), we adhere to the strict/original version of these principles with the main experiments into XOR and C-3D concept learning behavior.

### Pilot study results

As far as we are aware, presenting exemplars belonging to only one category to assess changes in modification behavior (i.e., concept completion) is novel to the current research and can be contrasted with many other unsupervised learning investigations using exemplars of at least two contrasting categories [4,50–52]. Though a novel method for assessing unsupervised learning behavior, we suggest it aligns with many real-world situations where we encounter successive examples of a categorical stimulus before encountering examples from contrasting or alternative categories (if at all). The current method thus permits an investigation of how observers may gradually extract relational information from a categorical stimulus without interference from one or multiple related categories of stimuli. Regardless of this new methodological approach, one may use results from these prior studies to hypothesize a greater likelihood of observing unsupervised learning behavior when observers have multiple and successive opportunities to extract the relational information inherent to the underlying categorical structure in our study. Indeed, some of these unsupervised learning investigations have found a greater propensity for learning when many exemplars belonging to the same category are presented in succession before exemplars from a contrasting category are presented [50–52]; but see [4] for an alternative account).

Importantly, we conducted a pilot study, which is reported fully in the S1 Appendix, where we assessed participants’ ability to complete partial 1D, XOR, and more complex C-3D concepts. This study involved a completely counterbalanced repeated-measures factorial design, whereby 1/3 of participants completed 1D problems before the other two types of problems, and vice versa for the other two orders. Although we did not observe increases in accuracy for most of the experimental conditions, we did see a significant increase in accuracy (and decrease in response times) for participants who completed the XOR category structure and the C-3D category structure first. However, because the design involved repeated measures, these two conditions were generally underpowered (*N* = 16 and 13 participants, respectively). Thus, a main goal of the current experiments is to further assess these two concept completion results by conducting a high-powered experiment involving participants only making modification decisions for one of these two relations. Each condition in the current experiment mirrored the design of the pilot study, which is reported at length in the supplemental materials, with a few minor methodological changes. First, we informed participants there was only one correct answer per trial and that it was possible to be perfectly accurate throughout the task. Thus, the current experiments more closely align with the unsupervised learning guidelines put forth by [1].

Second, we assessed XOR and C-3D concept completion across two isomorphic tasks for each participant. Participants completed partial concepts from category structures consisting of realistic clocks and realistic t-shirts [19]. We counterbalanced the order of presentation of these two stimulus types per participant. These experiments seek to replicate and extend upon the results of the pilot experiment by assessing changes in XOR and C-3D concept completion with substantially more statistical power and by assessing whether there is an increase in accuracy for the second (e.g., “t-shirt” stimuli) compared to the first task (e.g., “clock” stimuli). This extension involves participants transferring their learning from the first task to a novel set of stimuli in the second task. Observing a higher proportion of object selections for the second task (transfer) bolsters the argument that multidimensional unsupervised learning is occurring in these concept completion tasks.

Finally, we also seek to determine whether observers are more accurate in completing the XOR associated category structure compared to the C-3D associated category structure. As we describe further in the Discussion section, the non-parametric models derived from GIST [13,14,22,40] and GRIT [13–17,21] predict the XOR conceptual relation is easier to form than the C-3D conceptual relation. This predicted advantage arises as a natural consequence of observers extracting the relational information between the stimulus dimensions and recognizing that they only need to focus on two of the three stimulus dimensions for the XOR relation, compared to needing to focus on all three of the stimulus dimensions for the C-3D relation. Observing higher accuracy for the XOR relation compared to the C-3D relation provides converging evidence for applying the GIST and GRIT framework for understanding behavior in the current categorization tasks, and is consistent with many other empirical studies whose results have proven highly consistent with the predictions and explanations given by the GIST/GRIT framework (e.g., results related to human categorization, decision making, and informativeness judgments [11,17–23,40]).

As a final caveat, we again acknowledge that observing behavior consistent with completing the XOR or C-3D relation does not necessarily entail participants are forming these concepts or engaging in multidimensional unsupervised categorization. There are likely other alternative explanations for the behavior from the current tasks, some of which may imply little to no learning of interrelations between stimulus dimensions. In fact, we discuss an alternative explanation of our current results at length in our Discussions section (termed the “Minority rule” heuristic). However, this alternative explanation does not predict observing a difference in accuracy rates between XOR and C-3D behavior. Thus, observing a difference in accuracy between these two relations helps differentiate among competing accounts of behavior observed in our tasks.

## Method

### Participants

We recruited 48 and 46 participants from Introductory Psychology and Social Psychology courses at Marietta College for testing the XOR and C-3D concepts, respectively. Using G*Power 3.1, we conducted a power analysis (1−β=.95, α=.05,  one-tailed test) for detecting a medium effect size ($\hat{d}=0.5$) between performance across each participant’s first task with their performance in their second task and revealed a minimum sample size of 45 participants for this related-samples *t*-test. We achieved this sample size for both conditions, which is also substantially more participants needed to detect a medium effect size for a regression slope for each experiment at the same power level (*N* = 34).

Both the pilot study and this main experiment were approved by the Marietta College Human Subjects Committee (Protocol: 09272018−1). The procedures used adhere to the tenets of the Declaration of Helsinki and its later amendments or comparable ethical standards, and we received written informed consent from each participant. The data was collected between November 7^th^, 2018, and March 4^th^, 2022.

### Materials

We utilized a program written in MATLAB Version 2018b with Psychophysics Toolbox Version 3.0.14 and administered using Dell computers and monitors to display the object stimuli and to record response time and object selection information from participants. The raw and processed data are provided at the following Dryad website: https://doi.org/10.5061/dryad.ht76hdrtk.

The object stimuli were realistic clocks [11,19,45] varying over the three separable dimensions of *shape* (circular or square), *hand color* (white or black), and *number of edge tick marks* (few or many). Thus, there were a total of eight unique clock stimuli (e.g., circular clock with white hands and few edge tick marks). In addition, we also used realistic t-shirts [19] varying over the three separable dimensions of *color* (white or light beige), *neck type* (round or v-neck), and *number of horizontal lines* (one or three). Each experimental trial involved displaying three of the clock/t-shirt stimuli in random order before participants were presented with the group of five remaining clocks/t-shirts enclosed in a white box.

Depicted in Fig 2 are examples of the clock stimuli and an example of an experimental trial of the concept completion task.

The top row depicts the observation phase of the task whereby observers simply observed the sequential presentation of the object stimuli (realistic clocks). Each stimulus was presented for 3 s, followed by the presentation of a fixation cross for 1 s. The bottom row depicts the classification phase whereby observers selected the one object out of the five shown that they believed belonged next in the series. The category instance in the top row is an example of the 3_2_[3] – II structure and the most often chosen object in our experiments with this structure involved observers selecting the middle object (square clock with white hands and few tick marks), which results in completing the exclusive-or (XOR) concept associated with the 3_2_[4] – II category structure. The location of the presented objects were randomized per trial for both the 3-object sequence and the 5-object decision frame.

### Design

We created two experimental conditions by manipulating the between-subjects variable of *Category Structure*. For this variable, participants only made modification decisions for the 3_2_[3] – II type (*N* = 48) or for the 3_2_[3] – III type (*N* = 46). By repeatedly presenting instances of the 3_2_[3] – II category structure for participants to complete, we can determine whether participants engage in consistent and more accurate exclusive-or completion behavior by creating the 3_2_[4] type that results in the two-dimensional XOR relation (3_2_[4] – II). By repeatedly presenting instance of the 3_2_[3] – III category structure for participants to complete, we can determine whether participants engage in consistent and more accurate C-3D completion behavior by creating the 3_2_[4] type that results in the three-dimensional C-3D relation (3_2_[4] – VI). These two 3_2_[4] creations were the most common modifications to the presented 3_2_[3] structure in the pilot study (XOR: 68%, C-3D: 54%) and it is worthwhile to note that FR behavior is demonstrable for the latter structure, creating 3_2_[4] – IV, where C-3D sorting was predominantly observed (FR: 5%).

Fig 1 displays a Boolean cube visual representation of the tested category structures, whereby each corner on the cube represents one of the 8 possible object stimuli (clocks or t-shirts) that can be created with three binary-valued stimulus dimensions. The corners with black dots represent positive examples of the category where, for the current tasks, they were sequentially displayed in a random order before participants made their modification/completion decision based on the remaining five objects (corners enclosed by boxes with roman numerals creating one of the six 3_2_[4] category structures). The Boolean cube representation is particularly useful for delineating the different logical configurations, and increase in complexity, across the three 3_2_[3] structure types (compare the columns). Additionally, it is also useful for showing the possible resulting 3_2_[4] category structure types as participants decided how to complete the partial concepts throughout the tasks (compare proportions with their Roman numeral equivalent).

Each of the two tasks involved randomly assigning participants to complete the task with either the clock or t-shirt stimuli first. After completing the first task, they then completed the second task with the second set of stimuli. We added the second task with the different set of stimuli to assess whether participants would transfer any learned XOR or C-3D relational information. Because the new set of stimuli is different on the surface (t-shirts compared to clocks) but the same with respect to the structure, we predict seeing participants doing better in the second task if they are learning the structure of the category during the first task. If participants did not learn or retain any information about the XOR relation with the first set of stimuli (the clocks), then they should display a similar proportion of XOR accuracy at the start and throughout the second task with the second set of stimuli (the t-shirts).

Each of the two tasks involved participants making a series of 48 completion decisions for randomly presented instances of the specific category structure under consideration (3_2_[3] – II or 3_2_[3] – III). Thus, participants made a total of 96 completion decisions across the two isomorphic categorization tasks. Importantly, each 3_2_[3] – II or 3_2_[3] – III instance was sampled at random per trial from the set of all possible instances for that structure type. More specifically, there are 24 unique instances for type 3_2_[3] – II and 8 unique instances for type 3_2_[3] – III. For example, one participant may receive the following three instances from type 3_2_[3] – II for the clock stimuli to begin and end their first task:

*Instance 1*: circular/white/few → square/black/many → circular/white/many

*Instance 2*: circular/black/few → square/black/few → square/white/many

⁝

*Instance 48*: square/white/many → square/black/many → circular/white/few

For the first instance, completing the XOR concept with the “square/black/few” clock may lead to verbalizing “circular and white OR square and black”, whereas, completing the XOR concept for the second and 48^th^ instance may lead to verbalizing “black and few OR white and many” and “square and many OR circular and few”. This random presentation of the 3_2_[3] – II category structure instances across the duration of the task ensures that the dimensions relevant for solving the problem varied per trial for the XOR structures. That is, a property of completed XOR concepts is that one dimension becomes irrelevant for verbalizing the XOR logical rule and participants may learn to ignore this dimension (e.g., instance 1 = *number of edge tick marks*; instance 2 = *shape*; instance 48 = *hand color*). Importantly, there are more ways to present three of the eight stimuli at random that do not permit completing an XOR concept (e.g., 32 non-XOR instances associated with 3_2_[3] – I and 3_2_[3] – III), and we did not present any of these sequences for participants to complete if they were tasked with learning to complete the XOR concept.

Regarding the more complicated C-3D concept, a participant may receive the following three instances from type 3_2_[3] – III for the t-shirt stimuli to begin and end their first task:

*Instance 1*: light beige/v-neck/3 → light beige/round/1 → white/round/3

*Instance 2*: white/v-neck/3 → light beige/v-neck/1 → white/round/1

⁝

*Instance 48*: white/round/1 → light beige/round/3 → light beige/v-neck/1

For the first instance, completing the C-3D concept involves adding the “white/v-neck/1” t-shirt, whereas, completing the C-3D concept for the second and 48^th^ instance involve adding the “light beige/v-neck/3” and “white/v-neck/3” t-shirts, respectively. Logically, all three dimensions are important for completing the C-3D concept and this random presentation of instances helps to limit any memorization processes that may be influential when instances of this type are repeatedly presented to observers (a potential confound that we discuss in more detail in the Discussion section). Similar to above, there are more ways to present three of the eight stimuli at random that do not permit completing a C-3D concept (e.g., 48 non-C-3D instances associated with 3_2_[3] – I and 3_2_[3] – II), and we did not present any of these sequences for participants to complete if they were tasked with learning to complete the C-3D concept.

Finally, and as shown in the example instances above, the exemplar sequence presented to participants was independently randomized per trial for each participant. Additionally, the five objects that were possible additions to the presented category structure (see bottom pane of Fig 2) were also independently randomized per trial for each participant. Thus, randomization for each of the two tasks (XOR, C-3D) occurred at the following levels: Assignment to first task (clocks or t-shirts), Selection of 3_2_[3] category structure instances per trial, Order of three exemplars in presented sequence, and Order of five exemplars presented as choices for modifying the presented 3_2_[3] category structure.

### Procedure

Upon giving informed consent, participants were relayed verbal instructions regarding the task (see supplemental material for verbatim instructions). Most importantly, each participant was told that they would see a series of either clocks or t-shirts one-at-a-time and that they were to select the clock/t-shirt that should appear next in the series. Each participant was told there was one clock/t-shirt that should be selected on each trial (and was the correct answer) and that they could earn up to $3 (in addition to course credit) depending on how accurate they were at selecting clocks/t-shirts throughout the tasks. We paid $1 to each participant after reading the instructions to ensure they believed they would be paid (to maintain/increase motivation).

The brief training session was to familiarize participants with the task and first stimulus set and involved presenting 3 modification trials, one each of the three 3_2_[3] category structures (I, II, III) in a random order. After completing the training, participants began the first of two concept completion tasks (see Fig 2 for an example trial for the XOR category structure). The instructions were provided on-screen for participants to read before they started each of the two main tasks, and the order of which stimulus was presented first, clocks or t-shirts, was counterbalanced across participants. Participants completed a second 3-trial training session after the first main task to familiarize participants with the new stimulus set.

### Statistical analyses

As detailed above, our main between-subjects independent variable for each experimental condition is the partial concept being assessed (XOR or C-3D) across 12 blocks of 4 trials for each stimulus type (48 clock trials, 48 t-shirt trials). This division of the 48 trials into 12 blocks was arbitrary, as there was not rest for participants across trials. Our main dependent variables for each experimental condition are the proportion of choices that complete the XOR and C-3D concepts and the response times to make a choice for each trial. Using JASP version 0.19.3, we performed one-tailed simple linear regression analyses to determine if there were statistical increases in the proportions for completing the XOR and C-3D concepts across the 12 blocks of trials for each stimulus type (e.g., creating category structures 3_2_[4] – II and 3_2_[4] – VI, respectively). We also performed one-tailed simple linear regression analyses to determine if there were statistical decreases in the response times for completing the XOR and C-3D concepts across the 12 blocks of trials for each stimulus type. For regression analyses that failed to meet the assumption of linearity, we report simple regression results where the independent variable (Block) is log-transformed and we denote those results with a “log” subscript.

Also using JASP version 0.19.3, we conducted one-tailed related-samples *t*-tests to determine if there were a 1) statistically higher proportion of XOR or C-3D concept completions in each participants’ second task compared to their first task (evidence of transfer of learning) and 2) statistically lower response times for XOR and C-3D concept completions in each participants’ second task compared to their first task (evidence of transfer of learning). We conducted two-tailed *t*-tests to assess whether there was a statistical difference in XOR and C-3D concept completion for the first block of trials of the second task compared to the last block of trials of the first task. Lastly, we conducted a Bayesian binomial test with a Beta prior and posterior distribution and a Bernoulli likelihood function for each concept (XOR, C-3D) where we compared the proportion of participants who had more XOR or C-3D concept completions in their second task to the baseline proportion for representing no transfer of learning behavior across participants (e.g., prior distribution parameter centered at.5).

Finally, we conducted 2 x 12 mixed-ANOVAs with Relation (XOR, C-3D) as a between-subjects factor and Block (12 blocks of 4 trials each) as a within-subjects factor to assess whether there were statistical differences in proportion of XOR and C-3D completions between participants across their two tasks. Recall, non-parametric models derived from GIST and GRIT predict observing a statistically higher rate of XOR completions compared to C-3D completions.

## Results

In what follows, we report both proportion of correct decisions and the average response times to make decisions for both experimental conditions. Results reported for response times involved removing response times that were more than 2 SD’s away from the mean for a block of trials. Ultimately, this results in removing 4.8% and 6% of the response times for the two XOR tasks, and 4.8% and 4.4% of the response times for the two C-3D tasks. In the case of repeated-measures *t*-test results, we performed a listwise deletion procedure before analyzing response times.

### Two-dimensional exclusive-or concept (modifying 3_2_[3] – II)

Replicating the pilot study, completing the XOR concept was the predominant completion behavior for both the first and second tasks, with all but one participant (98%) choosing the object consistent with this conceptual relation across both tasks most often. We analyzed performance across 12 blocks of 4 trials each for each of the two tasks for these 47 participants. Thus, the following results represent statistical analyses conducted across a total of 4512 concept completions, further separated as 2256 concept completions for each of the two tasks.

Among these participants, there was a statistical increase in XOR completions during the first categorization task, $F(1,10)=37.12,p<\mathrm{.001},\;R^{2}=\mathrm{.79},\;one-tailed$. Upon assessing each type of stimulus separately, we found a statistical increase in XOR completions for each stimulus type (clocks: $F(1,\;10)=4.58,\;p=\mathrm{.029},\;R^{2}=\mathrm{.31},\;one-tailed$; t-shirts: $F(1,\;10)=30.05,\;p<\mathrm{.001},\;R^{2}=\mathrm{.75},\;one-tailed$). Importantly, this increase in XOR completions was accompanied by a statistical decrease in response times, $F_{\log}(1,10)=73.66,p<\mathrm{.001},\;R_{\log}^{2}=\mathrm{.88},\;one-tailed$. Again, assessing each type of stimulus separately reveals a statistical decrease in response times for each stimulus type (clocks: $F_{\log}(1,\;10)=33.89,\;p<\mathrm{.001},\;R_{\log}^{2}=\mathrm{.77},\;one-tailed$; t-shirts: $F(1,\;10)=22.34,\;p<\mathrm{.001},\;R^{2}=\mathrm{.69},\;one-tailed$). These results replicate the results reported in the pilot study, with substantially more statistical power, and suggest that two-dimensional unsupervised learning of the XOR conceptual relation occurred when the instructions stressed either consistency (pilot) or accuracy (current experiment).

**Fig 3:**
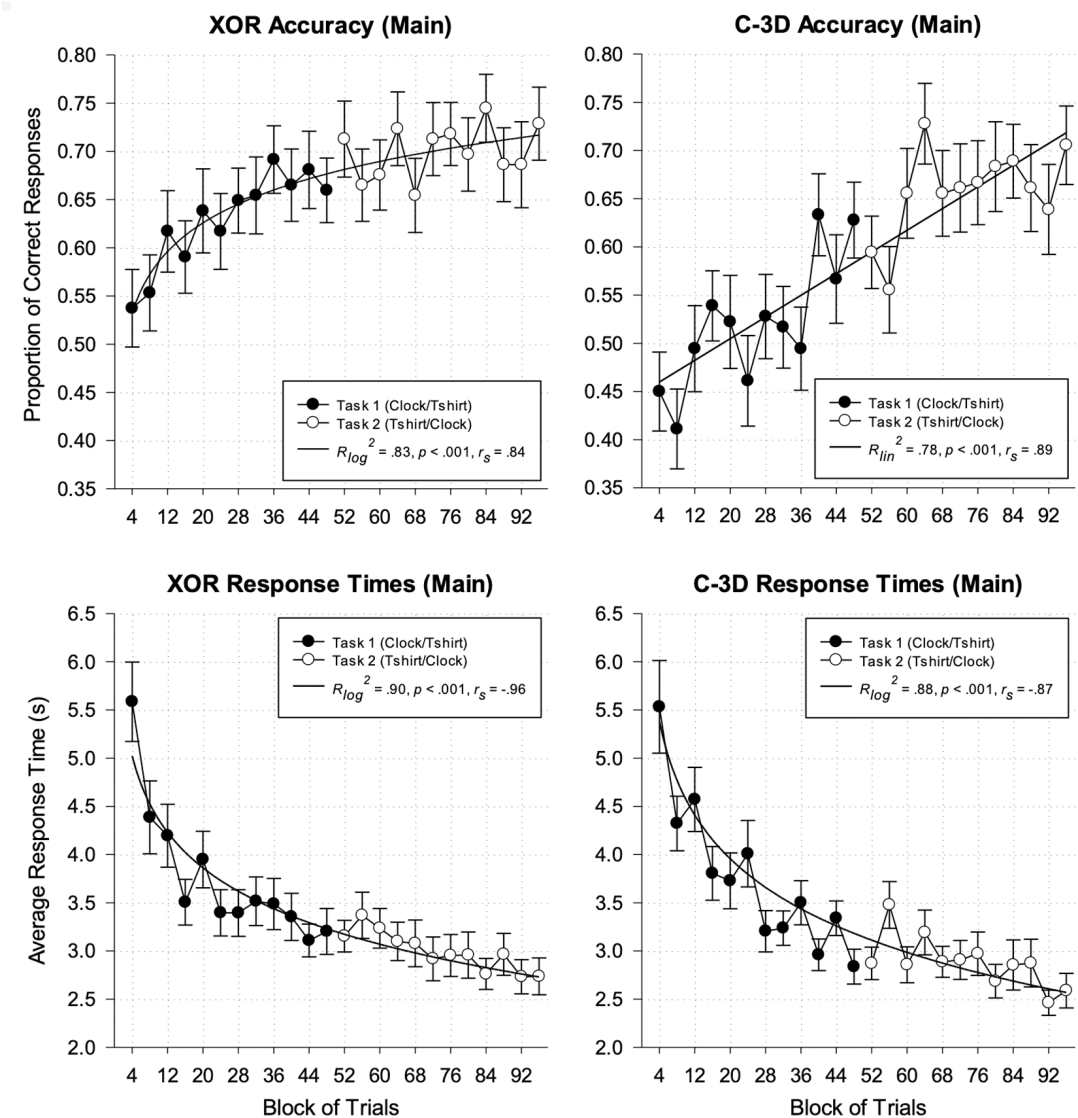
Aggregate classification and response time data associated with completing the partial exclusive-or (XOR) and complex three-dimensional (C-3D) Boolean concepts.

This pattern of results did not hold for the second task. More specifically, there was no statistical increase in XOR completions, $F(1,10)=1.08,\;p=\mathrm{.16},R^{2}=\mathrm{.10},\;one-tailed$. This lack of a statistical difference in XOR completions during the second task held for both stimulus types (clocks: $F(1,10)=1.44,\;p=\mathrm{.13},\;R^{2}=\mathrm{.13},\;one-tailed$; t-shirts: $F(1,10)=\mathrm{.02},\;p=\mathrm{.44},\;R^{2}=\mathrm{.002},\;one-tailed$). However, there was a statistical decrease in response times, $F(1,10)=40.62,\;p<\mathrm{.001},\;R^{2}=\mathrm{.80},\;one-tailed$, which held for both types of stimuli (clocks: $F(1,10)=8.67,\;p=\mathrm{.008},\;R^{2}=\mathrm{.46},\;one-tailed$; t-shirts: $F(1,10)=5.15,\;p=\mathrm{.024},\;R^{2}=\mathrm{.34},\;one-tailed)$. Despite the lack of change across the second task, the proportion of XOR completions (M=.70, SD=.15) was statistically higher than in the first task (M=.63, SD=.13), $t(46)=4.05,\;p<\mathrm{.001},\;\hat{d}=0.59,\;95\;\%\;CI:\;[0.28,\;0.90],\;one-tailed$, and the response times for the second task were statistically shorter (M=2.82 s, SD=0.74 s) than in the first task (M=3.61 s, SD=0.96 s), $t(36)=-6.19,\;p<\mathrm{.001},\;\hat{d}=-1.02\;[-1.41,\;-0.61],\;one-tailed$. Both results, accompanied by the statistically-significant regression analyses using logarithmic functions reported in the left column of Fig 3, suggest that learning of the XOR relation occurred predominantly in the first task and was successfully transferred to the new type of stimulus in the second task.

Shown in Fig 3 are the proportion of classification decisions resulting in completing an exclusive-or concept (top left pane) and the average response times to make such XOR completions (bottom left pane) across participants of the experiment. Also shown are the proportion of classification decisions resulting in a completing a complex-3D concept (top right pane) and the average response times to make such C-3D completions (bottom right pane) across participants of the experiment. Standard error bars and results from regression analyses using linear and logarithmic functions also provided in each plot.

**Fig 4:**
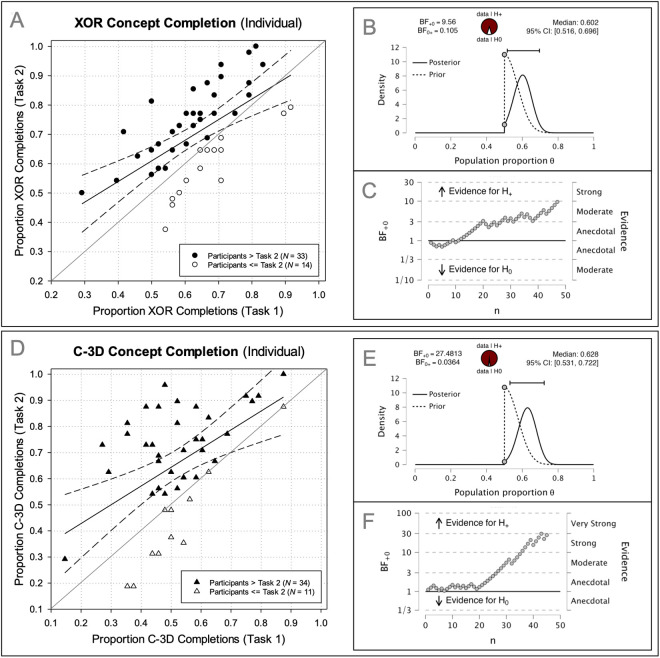
Transfer of learning the partial exclusive-or (XOR) and complex-3D (C-3D) Boolean concepts across experimental stimuli.

Further supporting the interpretation of transfer is the result that the proportion of XOR completions was not statistically different when comparing the last block of trials of the first task (M=.66, SD=.23) with those of the second task (M=.71,SD=.27), $t(46)=-1.01,\;p=\mathrm{.32},\;\hat{d}=-0.15\;[-0.43,\;0.14],\;two-tailed$. Additionally, the leftmost scatterplot (labeled A) in Fig 4 displays the proportion of XOR completions for each participant for their first (*x-axis*) and second (*y-axis*) task. Data points above the diagonal represent more XOR completions in the second compared to the first task (33/47 participants). Statistically, a Bayesian binomial test with a prior (θ~beta(24, 24)) centered at.5 and a Bernoulli likelihood function revealed evidence for more than 50% of participants performing better in the second task compared to the first task, ${BF}_{10}=11.53,\;Med=\mathrm{.61},\;[95\;\%\;CI:\mathrm{.52},\mathrm{.70}]$. The prior was motivated by the notion that no difference in performance across the first and second task would indicate half the participants performing worse (*N* = 24) and half performing better (*N* = 24). In other words, we specified this conservative prior to be consistent with the pattern of previous unsupervised learning results exhibiting a lack of multidimensional unsupervised learning behavior. Although specifying a uniform prior would lead to a posterior that more closely aligns with the current data, this more conservative prior begins with an assumption that there is no transfer in performance between the first and second task. These results demonstrate successful transfer of the XOR concept to the second concept completion task. The additional Bayesian analysis graphs provided in plots B and C of Fig 4 display important metrics regarding the estimated parameter for proportion of participants exhibiting more XOR completions in the second compared to the first task (panel B) and the stability of the statistical result for sample sizes less than we obtained (panel C).

Displayed in the top row (A) of Fig 4 are the proportion of XOR completions per participant across their two concept completion tasks, whereas the bottom row (D) displays the proportion of C-3D completions per participant across their two concept completion tasks. The plots directly to the right of these two plots (B, C, E, F) reveal the results of a Bayesian binomial test (effect size, stability of result across different sample sizes) with a prior specified as θ~beta(24, 24) and θ~beta(23, 23), respectively. Each prior was motivated by the notion that no difference between the first and second tasks should equate to half of the participants performing worse in their second task and half of the participants performing better in their second task.

### Three-dimensional complex-3D relation (modifying 3_2_[3] – III)

Completing the C-3D concept was the predominant completion behavior for both the first and second tasks, with all but four participants (11%) choosing the object consistent with completing this concept across both tasks. Only 1 participant selected a different object more often for each task and this participant was removed from further analysis. Thus, the following results represent statistical analyses conducted across a total of 4320 concept completions, further separated as 2160 concept completions for each of the two tasks.

We analyzed performance across 12 blocks of 4 trials each for each of the two tasks. Among these participants, there was a statistical increase in C-3D concept completions for the first categorization task, $F(1,10)=16.40,p=\mathrm{.001},\;R^{2}=\mathrm{.62},\;one-tailed$. Upon assessing each type of stimulus separately, we found a statistical increase in C-3D completions for each stimulus type (clocks: $F(1,\;10)=10.71,\;p=\mathrm{.004},\;R^{2}=\mathrm{.52},\;one-tailed$; t-shirts: $F(1,\;10)=8.06,\;p=\mathrm{.009},\;R^{2}=\mathrm{.45},\;one-tailed$). Importantly, this increase in C-3D completions was accompanied by a statistical decrease in response times as the task progressed, $F(1,10)=33.87,p<\mathrm{.001},\;R^{2}=\mathrm{.77},\;one-tailed$. Again, we assessed each type of stimulus separately and found a statistical decrease in response times for each stimulus type (clock: $F(1,\;10)=22.31,\;p<\mathrm{.001},\;R^{2}=\mathrm{.69},\;one-tailed$; t-shirts: $F(1,\;10)=34.55,\;p<\mathrm{.001},\;R^{2}=\mathrm{.78},\;one-tailed$). These results replicate the results of the pilot study, with substantially more statistical power, and suggest that learning the three-dimensional C-3D relation occurred when the instructions stressed either consistency (pilot) or accuracy (current experiment).

This pattern of results held, but was not as strong, for the second task. More specifically, there was still a statistical increase in C-3D completions, $F(1,10)=3.65,\;p=\mathrm{.043},R^{2}=\mathrm{.27},\;one-tailed$, and a statistical decrease in response times, $F(1,10)=8.95,\;p=\mathrm{.007},\;R^{2}=\mathrm{.47},\;one-tailed$. However, the increase in C-3D completions occurred for the clock stimuli, $F(1,10)=5.66,\;p=\mathrm{.019},\;R^{2}=\mathrm{.36},\;one-tailed$, but not for the t-shirt stimuli, $F(1,10)=0.25,\;p=\mathrm{.32},\;R^{2}=\mathrm{.02},\;one-tailed$. The decrease in response times occurred for both types of stimuli (clocks: $F(1,10)=8.3,\;p=\mathrm{.008},\;R^{2}=\mathrm{.45},\;one-tailed$; t-shirts: $F(1,10)=6.87,\;p=\mathrm{.013},\;R^{2}=\mathrm{.41},\;one-tailed$). Finally, the proportion of C-3D completions for the second task (M=.66, SD=.21) was statistically higher than in the first task (M=.52, SD=.15), $t(44)=5.00,\;p<\mathrm{.001},\;\hat{d}=0.75\;[0.41,\;1.07],\;one-tailed$; and the response times for the second task were statistically shorter (M=2.55 s, SD=0.73 s) than in the first task (M=3.20 s, SD=0.80 s), $t(28)=-6.61,\;p<\mathrm{.001},\;\hat{d}=-1.23\;[-1.71,\;-0.74],\;one-tailed$. Both results, accompanied by statistically-significant regression analyses using linear and logarithmic functions reported in the right column of Fig 3, suggest that learning of the C-3D relation occurred predominantly in the first task and was successfully transferred to the new type of stimulus in the second task.

Further supporting the interpretation of transfer is the result that the proportion of C-3D completions was not statistically different when comparing the last block of trials of the first task (M=.63, SD=.26) with those of the second task (M=.59,SD=.25), $t(44)=0.70,\;p=\mathrm{.49},\;\hat{d}=0.11\;[-0.19,\;0.40],\;two-tailed$. Additionally, the leftmost scatterplot (labeled D) in Fig 4 displays the proportion of C-3D completions for each participant for their first (*x-axis*) and second (*y-axis*) task. Data points above the diagonal represent more C-3D completions in the second compared to the first task (35/45 participants). Statistically, a Bayesian binomial test with a prior (θ~beta(23, 23)) centered at .5 and a Bernoulli likelihood function revealed evidence for more than 50% of participants performing better in the second task compared to the first task, ${BF}_{10}=34.49,\;Med=\mathrm{.63},\;[95\;\%\;CI:\mathrm{.54},\;\mathrm{.73}]$. These results demonstrate successful transfer of the C-3D learning to the second concept completion task. The additional Bayesian analysis graphs provided in plots E and F of Fig 4 display important metrics regarding the estimated parameter for proportion of participants exhibiting more C-3D completions in the second compared to the first task (panel E) and the stability of the statistical result for sample sizes less than we obtained (panel F).

### Comparing completion behavior between XOR and C-3D relations

We conducted a 2 x 12 mixed-ANOVA with Relation (XOR, C-3D) as a between-subjects factor and Block (12 blocks of 4 trials each) as a within-subjects factor to assess whether there were statistical differences in proportion of XOR and C-3D completions between participants across their first task. In addition to the significant main effect for Block and no statistical interaction, there was a statistical main effect for Relation, $F(1,\;90)=14.48,p<\mathrm{.001},\omega^{2}=\mathrm{.07}$. Post-hoc comparisons with Holm corrections applied revealed eight of the twelve comparisons to be statistically significant, each supporting the alternative hypothesis that the proportion of XOR completions were statistically greater than the proportion of C3D completions across participants, $Med\;t(90)=2.25,\;Med\;p_{Holm}=\mathrm{.026},\;Med\;Cohen^{\prime }s\;d=0.47$. Though these eight comparisons were statistically significant, all twelve comparisons involved a completion rate for XOR relations being directionally higher than the completion rate for C-3D relations. Thus, we observed differential creation of these two categories whereby the XOR categories (3_2_[4] – II) were easier to form for participants for their first task compared to the C-3D categories (3_2_[4] – VI). This result is consistent with non-parametric model predictions derived from GIST and GRIT.

The second 2 x 12 mixed-ANOVA to assess whether there were statistical differences in proportion of XOR and C-3D completions between participants across their second task revealed no statistical interaction and only a statistical main effect for Block, $F(11,\;990)=2.04,p<\mathrm{.022},\eta_{p}^{2}=\mathrm{.02}$. Upon collapsing across the Relation factor, there is weak evidence of an increase in accuracy rates for the second task and (visually) it seems to be mostly driven by increases across the first four blocks of the second task for the C-3D relation. Though this result does not support the prediction by GIST and GRIT, it does indicate that participants may have reached a performance ceiling for each of the two stimulus relations by the middle of the second task.

### Creating 3_2_[4] structure types

**Fig 5:**
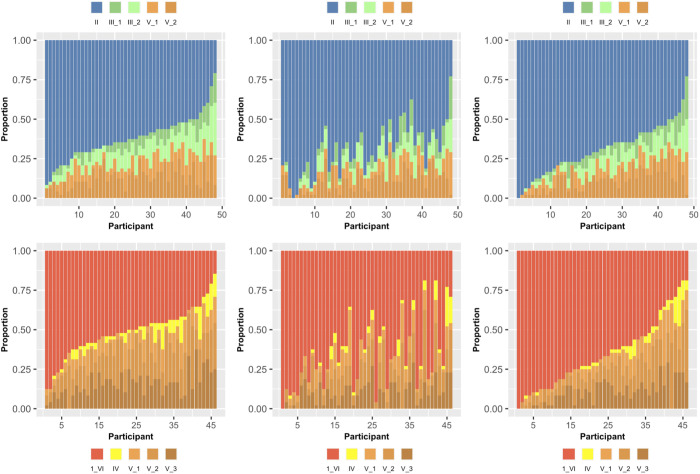
Analysis of partial exclusive-or (XOR) and complex-3D (C-3D) concept completion among participants.

In addition to assessing the presence of multidimensional unsupervised learning via XOR and C-3D concept completions, Fig 5 displays the differential creation of the 3_2_[4] structure types across these two experiments for each of the participants. This analysis of how often participants created each of these six category structures, which may be of broad interest to researchers who have investigated serioinformative and parainformative learning of these categories over the years [12,40,22,36,38,39,41,42,44,53,54]. Regarding the use of categorical stimuli defined by separable dimensions, the often replicated concept learning difficulty ordering of these six structures is I < II <[III, IV, V] <VI (in terms of increasing number of classification errors). Currently, our use of “XOR” refers to category structure type II, “C-3D” refers to category structure type VI, and “FR” refers to category structure type IV.

Individual-level creation of the 3_2_[4] structure types across the two structure types (3_2_[3] – II and 3_2_[3] – III) tested in the current experiment is shown in Fig 5. There are a different set of participants for the top row (XOR sorting) compared to the bottom row (C-3D sorting). For each row, the participants are ordered, left to right, from the most to least accurate in creating 3_2_[4] – II (top row) and 3_2_[4] – VI (bottom row). The order of participants for the second column maintains this ordering of accuracy; whereas, the order of participants for the third column reflects most to least accurate for the second experiment utilizing the novel set of stimuli (either clocks or t-shirts). Note that XOR and C-3D sorting are represented across the plots as types II (blue) and VI (red orange), respectively.

Each unique bar color across the two rows equates to one of the five possible choices for each of the two conditions of the concept completion task (XOR = top row = blue bars; C-3D = bottom row = red-orange bars). For each row, we sorted participants based on their XOR or C-3D concept creation rate (i.e., creating 3_2_[4] – II and 3_2_[4] – VI, respectively), with those achieving highest rates being represented as the leftmost bars for the plots in the first column of each row. This order was preserved for the plots in the second column, permitting an analysis of how being accurate in the first task is related to being accurate in the second task. Finally, the rightmost column removes the linkage between performance across participants’ first and second task by displaying each participant’s performance for their second task after sorting for XOR or C-3D concept completion (e.g., the most accurate for the second task are the leftmost bars in third column).

As shown in Fig 5, the second easiest to learn 3_2_[4] category structure (type II; row 1: blue bar) was created most often during the experiment with 66% of choices consistent with the XOR relation; whereas the most difficult to learn 3_2_[4] category structure (type VI; row 2: red-orange bar) was created most often during the experiment with 58% of choices consistent with the C-3D relation. FR conceptual behavior was virtually non-existent (yellow bars in plots in bottom row). Participants who were more accurate creating type II in the first task were also more accurate in the second task, r(46)=.69,p<.001, 95% CI:[.50, .81]. Additionally, participants who were more accurate creating type VI in the first task were also more accurate in the second task, $r_{s}(46)=\mathrm{.45},p<\mathrm{.001},\;[\mathrm{.19},\;\mathrm{.66}]$.

## General Discussion

A primary goal of the current work was to assess whether observers complete multidimensional conceptual relations without feedback when the compositional nature of the stimulus dimensions and the categorization task are simplified. We suggest that prior unsupervised learning investigations utilizing continuous valued or multi-valued stimulus dimensions, and categorization tasks theoretically more complex than necessary for observers to demonstrate more complex types of unsupervised learning behavior, may have inadvertently made it more difficult to observe such phenomena. Upon simplifying the stimulus dimensions and the categorization task to present the minimum amount of category exemplars with our three-dimensional category structures, we found observers gradually became more consistent and accurate in selecting objects that completed a two-dimensional exclusive-or logical relation and a three-dimensional complex logical relation. Further supporting our claims of multidimensional concept completion are the combinations of increases in consistency and accuracy being accompanied by decreases in response times to make such decisions.

We contend our current experimental methodology satisfies the four constraints put forward by [1] for investigating unsupervised *learning* behavior, and not merely *preference* behavior. Although the Boolean structures do not necessarily involve different clusters of exemplars in multidimensional space, they are logically distinct and arguably vary in their levels of coherence. In fact, our results with XOR and C-3D concept completion rates suggest that the methodological constraint of ensuring separate clusters of exemplars is unnecessary for demonstrating multidimensional unsupervised learning behavior. Additionally, this latter learning result is of particular interest given that participants could have selected the prototype of the presented three-object sequence to form the linearly separable type IV of the 3_2_[4] structure family. More importantly, however, is the fact that these same category structures have been employed across a myriad of concept learning experiments for over 60 years, highlighting the limiting constraint of category structures “needing” to occupy clusters in multidimensional stimulus space [40,22,36,38,39,44,55]. In short, we suggest there is currently no objective reason for requiring the constraint that category structure must occupy clusters in multidimensional stimulus space.

Regarding practice, observers made a series of 48 concept completions for each task and had ample opportunity to uncover the underlying category structure. Our results indicate that repeated and minimal (in terms of number of category exemplars) exposure to the entire underlying category structure facilitated multidimensional unsupervised learning. This implication is vastly different to how practice was operationalized and instantiated in prior unsupervised learning experiments whereby the entire category and contrasting category were intermixed and presented only once during the observation blocks [1,2]. Finally, we observed multidimensional learning when the instructions stressed either consistency (pilot) or accuracy (current study), with the former excluding the prescription by [1] to notify observers that an underlying category structure exists. Thus, the original prescription stressing accuracy and notification of an underlying structure may facilitate unsupervised learning behavior but may not be strictly necessary to observe such behavior.

### Accounting for unsupervised learning behavior

A secondary goal of the current work involves accounting for the multidimensional learning behavior when feedback is not provided to aid such learning. Do existing theories and formal models of unsupervised learning behavior account for the current results? Although these models are scarce in the literature when compared to their supervised counterparts, many require a sequential presentation and classification of exemplars into one of two categories, which does not align with the design of the current experiments [56–58]. Certain models, such as the *simplicity model* [24,25,59,60] or one derived from *generalized representational information theory* (GRIT; [13–17,21]) do not make such assumptions and may be applied to better understand the incremental learning that occurred in the current tasks.

### The simplicity model

This model is derived from a theory of unsupervised categorization behavior that is consistent with the FR tenets of [28], specifying that observers seek to maximize similarity between exemplars belonging to a category while minimizing similarity between exemplars belonging to contrasting categories [24,59,60]. The simplicity model assumes observers seek to create the most “intuitive” groupings of stimuli, and this is mathematically calculated using principles from information theory [61,62]. A caveat, however, needs mentioned with the current application of the simplicity model. Namely, that the current sequential presentation of exemplars does not align with the types of situations for which the simplicity model was designed to account. More specifically, one may argue that the simplicity model is best applied to more “perceptual” types of grouping tasks where the notion of “intuitive” and “unintuitive” groupings of simultaneously presented stimuli may inform conceptual processes. Keeping this caveat in mind, we primarily see the current application of the simplicity model as a way to assess how well computational predictions that align with family resemblance principles account for the current results.

Regarding the current tasks, the simplicity model accurately accounts for the prevalent 1D concept completions when observers completed the 3_2_[3] – I category structure (pilot). However, the model is unable to account for the unsupervised learning of the XOR and the C-3D concept completions. The model cannot account for the former because it predicts type III of the 3_2_[4] structures is the most intuitive classification when category structure 3_2_[3] – II is being completed. Regarding the latter, the model predicts type IV of the 3_2_[4] structures is the most intuitive classification when category structure 3_2_[3] – III is being completed. Interestingly, the model predicts category type II (XOR) and category type VI (C-3D) of the 3_2_[4] family of category structures are the least intuitive classifications when they are possible to create. Thus, it seems across most experimental trials, observers were neither maximizing similarity between exemplars in each category, nor were they minimizing similarity between exemplars of contrasting categories.

### The generalized representational information theory model

Generalized Invariance Structure Theory (GIST) posits a specific kind of pattern detection mechanism named “invariance detection” as the basis for concept formation and other key cognitive capacities. The mechanism extracts from environmental and mental stimuli key information in the form of a meta-representation referred to as an “ideotype”. Ideotypes determine the perceived degree of difficulty of a concept and provide the substrate for prototype formation and rule formation as derived concept representations. The equation describing the perceived degree of learning difficulty is given in S2 Appendix. For a mathematical description of the process of invariance detection see the Appendix of [22] or [13,14,40]. Here we only give a brief intuitive explanation of the core ideas.

To begin, invariance detection consists of a series of cognitive subprocesses or mechanisms, starting with a fundamental mechanism known as “dimensional binding”. The mechanism involves systematic rapid attention shifting from one dimension to another accompanied by sustained and deliberate “unattending” or “suppression” with respect to each dimension. Ultimately, the described process results in the detection of categorical/concept invariants on a per dimension basis. These invariants are the object exemplars that determine which features (and more generally, dimensions) of the categorical stimulus should be used in rule formation (see [13–15,40,63] for a detailed explanation). The proportion of categorical invariants of a categorical stimulus are then represented as an “ideotype” in psychological ideotype space (i.e., ideotypes contain the structural information necessary to form concepts and every concept structure has a corresponding ideotype).

Ideotypes act as precursors for and inform the construction of other representations such as rules and prototypes. As meta-representations, ideotypes are also useful for computing learning difficulty judgments and other types of judgments per higher level rules such as the law of invariance at the core of GIST. The overall degree of invariance associated with a category is computed via the Euclidean metric which determines the distance of any ideotype from the reference zero point in the coordinate space (i.e., the zero ideotype). Intuitively, the overall degree of invariance of a category can be understood as the overall degree of logical coherence of the category as determined by its overall proportion of invariants across dimensions. Logical coherence refers to how strongly the feature values of dimensions are interrelated or logically interdependent as determined by the specific form of invariance detection described in GIST (namely, s-invariance; see [13] for the formal details). Accordingly, the zero ideotype represents maximal incoherence or minimal logical coherence. This is equivalent to a state of complete independence between the object stimuli of a categorical stimulus requiring the use of all dimensions to predict category membership.

The core law of invariance in GIST is a statement that relates degree of subjective learning difficulty to the degree of overall categorical invariance or coherence as detected in a categorical stimulus via the binding mechanism and ideotype formation (see the technical appendix for the mathematical description). A plethora of experiments have shown the law to be effective in predicting, without free parameters and/or with a few free parameters, the degree of learning difficulty associated with many category structures including structures based on integral dimensions, such as key categories involving color dimensions [22].

Generalized Representational Information Theory (GRIT), on the other hand, is a theory of subjective information that is based on the invariance detection principles described and posited in GIST and that purports to quantify information subjectively. The basic idea is that information as a subjective construct is determined by categorical invariance or logical coherence and categorical complexity rather than by the (in)probability of events as in the misguided application of Shannon and Weaver information [62]. The basic measure at its core, known as representational information, measures the quantity of relative information associated with a subset of a category as a discrete rate of change (proportional or as a percent) in the perceived subjective complexity (operationalized empirically as learning difficulty) of the category as the subset is suppressed, where the subset typically consists of one element. Accordingly, what is being measured is the contextual impact that a particular exemplar(s) has on the overall complexity and logical coherence of the category. The basic equation for computing the degree of representational information of a subset of a categorical stimulus is given in the technical appendix.

**The Need for Informativeness as a function of Coherence.** Among the basic meta-principles underlying GIST and GRIT that place the constructs of complexity and invariance at their helm, there are two which, along with one of the invariance detection principles referred to as the *structural equilibrium principle*, may explain the results from our experiments (for details see chapter 6 of [14]). The first is that humans have a need and innate tendency to detect logical coherence as characterized by invariance, the second is that humans have a need and innate tendency to minimize complexity (i.e., to simplify perceived complexity in their internal/external environments). On the other hand, the structural equilibrium principle states that the perceived complexity of categorical stimuli is moderated by the proportion of fully diagnostic dimensions as determined by their corresponding ideotypes. For example, one of the most compelling results from the current experiment is that a majority of participants on average selected the object that completes a type six category structure (3_2_[4] – VI; C-3D concept completions). This structure is devoid of logical coherence from an invariance standpoint (as defined in GIST) but the propensity for its completion is due to structural equilibrium. Specifically, the slight reductions in subjective complexity as indicated by the existence of fully diagnostic dimensions in all the components of the ideotypes corresponding to the 3_2_[4] – VI and 3_2_[3] – III structures accounts for this interesting phenomenon. Indeed, these contributions are enough to moderate the rate of change in subjective complexity as mathematically determined by the representational information measure in GRIT.

Accordingly, for structures not in total equilibrium, we also found that, on average, selected items by participants in each experiment were indeed consistent with predictions made by the representational information measure of GRIT in that subjects would select the object stimulus that, upon being inserted in either the 3_2_ [3] – I or 3_2_ [3] – II category sequence, would increase its logical coherence while lowering its complexity (creating 3_2_ [4] – I and 3_2_ [4] – II, respectively). Table 1 shows the degree of representational information expressed by each of the selected object stimuli in our experiment with respect to their category structure of origin (“GRIT-NPE Predictions”). Note that, overall, these values consistently reflect the selection of object stimuli from the set of alternatives (“Object Proportions”) and the goodness-of-fit statistics provided in the rightmost column (proportion of variance accounted, Spearman rho) across all comparisons indicate a strong relationship between the GRIT predictions and the observed data.

**Table 1:** Comparison of GRIT Model Predictions and Classification Decisions Across the Three Experiments.

Boolean Structure	Expt.	Possible Modification Decisions	GRIT-NPE Predictions^a^	Object Proportions	$R^{2}$ $(r_{s})$
3_2_[3] – I (**1D**)	Pilot	001, 011, 101, **110**, 111	(−.49, −.49, −.49,**.57**, −.42)	(.04,.05,.04,**.80**,.07)	.99 (.92)
3_2_[3] – II (**XOR**)	Pilot	001, 011, 100, **101**, 110	(−.38, −.45, −.38,**.09**, −.45)	(.08,.06,.10,**.68**,.08)	.99 (.87)
	Current	001, 011, 100, **101**, 110	(−.38, −.45, −.38,**.09**, −.45)	(.08,.05,.12,**.66**,.09)	.99 (.74)
3_2_[3] – III (**C-3D**)	Pilot	001, 010, 100, **101**, 111	(−.30, −.38, −.30, **−.25**, −.30)	(.13,.05,.13,**.54**,.16)	.65 (.92)
	Current	001, 010, 100, **101**, 111	(−.30, −.38, −.30, **−.25**, −.30)	(.08,.04,.13,**.58**,.16)	.61 (.89)

*Note*. SD = Single dimension partial concept, XOR = Exclusive-or partial concept, C-3D = Complex 3-dimensional partial concept.

^a^GRIT predicts observers will select the object that minimizes the subjective degree of concept learning difficulty of the resulting category (3_2_[4] category structure). Objects with the most positive rate of change value (bolded) for each of the three *Boolean* structures represent predictions for the current experiments (see [11,23] for an explanation).

### Potential limitations and concerns

Notwithstanding the current results and explanations thereof, there are potential limitations with the current work. First, it is unclear whether the multidimensional learning results associated with the C-3D concept completions were due to observers gradually uncovering the relational structure between exemplars (e.g., conceptual processes) or more to memory-related processes. The latter cannot be ruled out when considering that there are only two unique instances associated with creating the 3_2_[4] – VI structure type, compared to the 6 unique instances associated with creating both the 3_2_[4] – I and 3_2_[4] – II structure types. More specifically, the two instances associated with this structure type, where each set of three numbers represents a unique exemplar, are {000, 011, 101, 110} and {001, 010, 100, 111}. The current concept completion task with the 3_2_[3] – III category structure involved selecting one of these two instances and randomly presenting three of the four objects before observers made their completion decision. This results in only 8 unique combinations of 3 exemplars to present before observers make a completion decision (compare this to 24 unique combinations of 3 exemplars for the 3_2_[3] – I and 3_2_[3] – II concept completion tasks). Across participants, we observed learning across the 48 and 96 total concept completions; however, it is possible that observers recognized the repetition of specific category instances in the series of exemplars and (implicitly or explicitly) memorized the four exemplars for each of the above two instances. Indeed, this structure type is the most difficult to learn in supervised tasks and researchers have suggested that observers resort to rote memorization of category exemplars for this type in those tasks [38,39]. Although it may be possible to control for this possibility in the future, it is important to note that there was very little evidence of verbal descriptions given by participants that indicated memorization of exemplars (see Table S2 in the S3 Appendix). Notwithstanding, this smaller set of exemplar combinations coupled with their repeated presentation to observers makes it more difficult to disentangle memory-related influences when comparing XOR and C-3D concept completion behavior with the current tasks. Indeed, we thank an anonymous reviewer for the idea that some participants in the current tasks may have been using both a 1) less-efficient memorization-related strategy and 2) more-efficient “Minority-rule” heuristic (discussed shortly) for the C-3D structures. This combination may be less likely to be used with the XOR structures, which may then lead to more XOR concept completions (heuristic only) compared to the C-3D completions (memorization + heuristic). Future research should equate the number of these unique exemplar combinations across experimental conditions to further tease apart and determine the extent to which memorization-related processes may be impacting observers in the current experimental paradigm.

**Table 2:** Accuracy of 1D, XOR, and C-3D Relations for the Different Orders of the Within-Subjects Pilot Study.

Order (Relations)	1D	XOR	C-3D	First Task	Second Task	Third Task	Ordinal
1 (1D, XOR, C-3D)	.86	.72	.57	.86	.72	.57	1D > XOR > C-3D
2 (1D, C-3D, XOR)	.86	.81	.71	.86	.71	.81	1D > XOR > C-3D
3 (XOR, 1D, C-3D)	.67	.57	.45	.57	.67	.45	1D > XOR > C-3D
4 (XOR, C-3D, 1D)	.81	.63	.62	.63	.62	.81	1D > XOR > C-3D
5 (C-3D, 1D, XOR)	.79	.63	.37	.37	.79	.63	1D > XOR > C-3D
6 (C-3D, XOR, 1D)	.83	.71	.51	.51	.71	.83	1D > XOR > C-3D

Note. Participants completed 48 trials for each of the three relations. Samples sizes (*N* *=* *# of participants*) for the six orders are: 8, 9, 9, 9, 10, 9.

A second limitation with the current work is our lack of observing one-dimensional unsupervised learning behavior in our pilot study. Although we did see a predominance of this type of concept completion (80%) when observers were presented with the 3_2_[3] – I category structure, we did not see a statistical increase in this behavior as the task progressed. This failure to observe learning behavior with the simplest structure is perplexing and arguably makes it more difficult to isolate factors affecting unsupervised learning observed in previous sorting investigations and in our own [1,2,10]. Regardless, the consistency for observers to implement this one-dimensional behavior exceeded their ability to engage in XOR (68%, 64%) or C-3D concept completion behavior (54%, 58%), even when considering the learning gains reflected in the most accurate block of the current high-powered experiment with the XOR concept (74%). Thus, there seems to be a performance ceiling with the concept completion task, reflected with performance in the one-dimensional condition (e.g., 3_2_[3] – I structure type). Notwithstanding, we did observe perfect 1D concept completion for 2/54 participants. Worthwhile future research directions for increasing the likelihood of uncovering one-dimensional learning (e.g., observe performance below the performance ceiling) with the current tasks involves either shortening the length of time each object is presented during the observation period or increasing the number of stimulus dimensions composing the categorical stimuli. Additionally, using eye-tracking technology to better understand the attentional patterns underlying concept completion decisions is a promising future direction. Previous efforts using eye-tracking technology have proven useful in revealing interesting saccadic and fixation patterns during category learning [18,44], so expectations would be high using this approach.

One last issue concerns the extent to which observers in the current tasks are using “simple” heuristics for completing the presented categories, rather than mostly categorization or relational information processes, and whether these heuristics can be applied by considering each dimension in isolation. The latter implication is particularly important, as it has the potential to invalidate an interpretation of multidimensional unsupervised learning with the current tasks. We thank an anonymous reviewer for their exposition of this possibility and our description of their suggested heuristics is a paraphrasing from the reviewer. Regarding the 1D concept completion task, we can apply a heuristic that if all three presented objects share a value on a dimension (e.g., circular clock), then the next object in the series should also have this dimensional value. Regarding either XOR or C-3D concept completion, we can apply an iterative “Minority rule” heuristic that if two values on a dimension have been presented (two clocks are circular) and only one of the others has been presented (one square clock), then the next object in the series should also be a square clock. This second heuristic is iterative because it must be applied to all dimensions for it to successfully lead to behavior consistent with XOR or C-3D concept completion. When applied as mentioned, these two heuristics reproduce the observed pattern in the data for the current experiments and do so without consideration of how one dimension is interrelated to another dimension.

Although the second heuristic leads to behavior consistent with XOR and C-3D concept completion, its application does not lead to predicting a differential amount of XOR or C-3D concept completion. In other words, for this heuristic to reproduce the data, all three dimensions must be equally considered for both relations. The mixed ANOVA result showing that participants completed more XOR relations than C-3D relations cannot be explained by mere application of this heuristic. Additionally, if the heuristic is “simple” and involves “little effort (low computational complexity)” as suggested to us, then it is perplexing why we are seeing errors by participants on ~1/3 of trials for the XOR concept and ~3/5 of trials for the C-3D concept. Participants were given extensive practice with these partial concepts (96 trials), so we would expect many to become more proficient with the use of this heuristic if it is indeed simple and of “little effort (low computational complexity)” and we would expect more participants to verbalize using such a heuristic. However, as discussed in the verbal analysis section provided in Appendix B and shown in Tables S1 and S2 in the S3 Appendix, very few participants reported strategies or descriptions of their behavior that align with this heuristic (~6% per relation).

Interestingly, we can delve deeper into how extensive practice affects XOR and C-3D completion behavior and whether the behavioral patterns in the completion data are consistent with the heuristic. More specifically, using the heuristic we may predict that participants would display higher accuracy rates for either the XOR or C-3D relation if they just completed the other relation. Recall, the pilot study that we conducted was a completely counterbalanced within-subjects design whereby participants completed all three types of learning problems (1D, XOR, C-3D), which allows us to look into this prediction. We provide the average completion rates for each of these relations for each of the six conditions/orders in Table 2 (e.g., 1D-XOR-C3D, 1D-C3D-XOR, etc.). We do see a possible facilitative effect for completing the XOR relation (Order 2 = .81, Order 6 = .71) after immediately completing the C-3D relation first (Order 2 = .71, Order 6 = .51). However, each of these effects is also predicted by the GIST/GRIT framework as GIST predicts that the XOR relation is easier for participants to learn than the C-3D relation. More importantly, consistent with the GIST/GRIT framework and inconsistent with the heuristic is that we observed a higher accuracy rate for the XOR relation compared to the C-3D relation across all six participant orders (see “1D”, “XOR”, “C-3D”, and “Ordinal” columns in Table 2), which mirrors the ANOVA and post-hoc results we reported above. Finally, the rate of XOR completions does not appear to be different if participants first completed the 1D relation (Order 1 = .72) or C-3D relation (Order 6 = .71). We do not see much, if any, of a facilitative effect for completing the C-3D relation (Order 1 = .57, Order 4 = .62) if participants completed the XOR relation first (Order 1:.72, Order 4 = .63). Contrary to the heuristic prediction, participants seem to have higher C-3D accuracy after completing the 1D relation (Order 2 = .71) compared to the XOR relation (Order 4 = .62); though, this effect reverses when comparing Order 3 and Order 1. Admittedly, these results are post-hoc, likely underpowered, and a subset of all interesting comparisons of these relations within participants. However, taken together, they do not provide much evidence of a facilitative effect for using the heuristic across related problems (XOR, C-3D). Thus, overall, the data is more consistent with the mechanism described in GIST than with the heuristic.

Finally, to successfully apply either of these strategies/heuristics (whether verbally or implicitly), we suggest that observers need to assess the relationships between stimulus dimensions to recognize both the diagnostic value of the relevant dimensions and the redundant value of the other irrelevant dimensions. Thus, some participants may be using these heuristics (and possibly, verbalizing them), but that is consistent with the notion that observers first need to extract the diagnostic value of each dimension which is what the invariance-detection mechanism in GIST achieves. In other words, GIST as a theory utilizes more fundamental and intrinsic cognitive mechanisms and purports that its key representation, an ideotype, serves as a precursor for understanding how rule representations (and related, heuristics) come into existence in the first place. Thus, for observers to use the minority rule heuristic implies that they first need to determine how useful each dimension is for categorization via the dimensional binding cognitive mechanism described in GIST (for formal details, see [13,14]).

Further supporting this interpretation is the fact that the XOR categories are reducible to two fully diagnostic dimensions, where the third dimension can be fully ignored, whereas the C-3D categories involve all three dimensions being equally diagnostic. This reduction in complexity associated with the XOR categories is consistent with the currently observed reduced error rate for this relation compared to the C-3D relation for both the main experiments and the pilot experiment. Relating this behavior to GIST and GRIT, this earlier processing of relational comparisons between objects and stimulus dimensions leads to identification of the diagnostic or redundant value of each stimulus dimension, which affects subsequent categorization behavior or implementation of any decision heuristic. In sum, GIST provides a causal description of potential cognitive mechanisms underlying rule and heuristic development, which is a necessary step for understanding why observers resort to using the minority rule heuristic (if they do use it) compared to other possible heuristics.

We believe the current work takes some initial key steps that will stimulate and challenge readers to think about this domain in unprecedented ways. For example, a promising study idea relayed to us by an anonymous reviewer involves assessing concept completion behavior for partial XOR or C-3D concepts (2-objects instead of 3-objects, as was done currently) after participants have demonstrated unsupervised learning of the XOR or C-3D concepts. The available choices for participants in this proposed task involve participants selecting objects that are either consistent with the XOR or C-3D structure. Would participants be more likely to select the XOR-consistent objects compared to the C-3D consistent objects after just learning the XOR structural relation, and vice versa for the C-3D relation? The heuristic account does not predict a differential selection of XOR- or C-3D-consistent objects, whereas GIST/GRIT does predict differential selection of these objects depending on the context surrounding the learning problem. The current experimental paradigm is flexible to address this scenario and other interesting possibilities as they arise.

## Conclusion

In conclusion, we suggest that observers were engaging in multidimensional concept completion with a categorization task utilizing simpler types of multidimensional object stimuli and category structures (i.e., Boolean) than traditionally implemented in the literature. We believe these results reveal the utility of the current concept completion tasks, and Boolean structures more generally, for uncovering a range of differentially complex unsupervised learning behavior. Indeed, it is readily apparent how utilizing different Boolean structures than those currently tested helps tease apart limits of our conceptual systems to learn without the aid of corrective feedback. As such, these findings help to both differentiate and to conceptually integrate basic notions of supervised and unsupervised human and machine learning in the extant literature on categorization. Notwithstanding, categorization theories that propose to be general in nature should be tested to see how they account for the current results and for subsequent investigations using a similar task and Boolean structures. As purported above, GRIT provides a parsimonious account of the current learning results and the results from a myriad of prior unsupervised categorization tasks [6–8,11,23,64]. We look forward to future research investigations that further test limits of the information measure proposed in GRIT and from competing theories, and we hope the current work helps spur such relevant research.

## Supporting information

S1 Appendix Pilot study methodology and results. (DOCX)

S2 Appendix Brief synopsis of the law of invariance and RIT measure. (DOCX)

S3 Appendix Analysis of participant verbal responses during experiment debriefing. (DOCX)

S4 Appendix Full results of all regression analyses (coefficients, standard error, p-values, confidence intervals, goodness-of-fit). (DOCX)
