# Supplementary material for: Constructing concepts without feedback: An empirical investigation of how relational information affects multidimensional concept completion behavior in an unsupervised task
Source: PLoS One. 2025 Aug 7;20(8):e0328368. doi: 10.1371/journal.pone.0328368 (PMC12331049; doi:10.1371/journal.pone.0328368)
Supplement: S1 Appendix — (DOCX) [file pone.0328368.s001.docx]

**S1 Appendix**

**Pilot study method and results**

**Method**

**Participants**

We recruited 54 participants from Introductory Psychology courses and Social Psychology courses at Marietta College. Participants received partial course credit and a small monetary reward (described below) for their participation. Regarding ethical approval, all experiments reported herein were approved by the Marietta College Human Subjects Committee.

**Materials**

We utilized a program written in MATLAB Version 2018b with Psychophysics Toolbox Version 3.0.14 and administered using Dell computers and monitors to display the object stimuli and to record response time and object selection information from participants. The raw and processed data are provided at the following Dryad website: https://doi.org/10.5061/dryad.ht76hdrtk.

The object stimuli were realistic clocks (Doan, 2018; Doan & Vigo, 2016; Vigo & Doan, 2015) varying over the three separable dimensions of *shape* (circular or square), *hand color* (white or black), and *number of edge tick marks* (few or many). Thus, there were a total of eight unique clock stimuli (e.g., circular clock with white hands and few edge tick marks). Each experimental trial involved displaying three of the clock stimuli in random order before participants were presented with the group of five remaining clocks enclosed in a white box.

**Design**

We manipulated one within-subjects variable (*Structure Type*) that had three levels of variation. This variable involves systematically manipulating which three clocks are randomly presented such that the dimensional relationships between the three clocks adhere to a specific Boolean logical configuration. Currently, we ensured that each set of three clocks was a logical equivalent of one of three Boolean structure types (3_2_[3] – I, II, or III). We chose these three types because modification decisions, in terms of selecting an object stimulus to add to the presented 3_2_[3] type, results in participants creating one of the six 3_2_[4] structure types. The 3_2_[4] structure types have been extensively studied over the past 60 years from the standpoint of concept learnability in supervised and parainformative classification tasks (Anderson, 1991; Feldman, 2000; Nosofsky, Gluck, et al., 1994; Rehder & Hoffman, 2005; Shepard, Hovland, & Jenkins, 1961; Vigo, 2013). Much is known regarding the learnability among the six 3_2_[4] structure types and we suggest that the differential creation of these structure types across the current tasks when feedback is not provided adds a unique and valuable perspective to the unsupervised categorization literature.

The first structure type (3_2_[3] – I) involves presenting three clocks that all share a particular dimensional value (e.g., three circular clocks). For this type, participants can implement a one-dimensional “rule” (1D) by selecting the fourth circular clock among the five that are enclosed in the white box. Selecting this clock creates structure type I of the 3_2_[4] family. Participants consistently engaging in this behavior align with the results of previous serioinformative (Ashby et al., 1999; Colreavy & Lewandowski, 2008; Love, 2002) and parainformative (Doan & Vigo, 2016; Imai & Garner, 1965; Medin et al., 1987; Milton & Wills, 2004; Regehr & Brooks, 1995) unsupervised investigations. Alternatively, observers can also choose to engage in three-dimensional family resemblance (FR) sorting for this type by selecting the clock that maximizes its shared properties with the three circular clocks. Selecting this clock creates structure type IV of the 3_2_[4] family.

The second structure type (3_2_[3] – II) does not involve presenting three clocks that all share a particular dimensional value. As an example, we may present the following three clocks: {square/black/few 🡪 square/black/many 🡪 circular/white/few}. For this type, participants can implement a two-dimensional exclusive-or (XOR) rule by selecting the fourth circular clock with white hands (circular/white/many). Notice that by selecting this clock, participants are ignoring variation on the third dimension (few or many tick marks). Participants consistently engaging in this behavior align with the results of Love (2002) with structure type II of the 3_2_[4] family and with recent parainformative sorting experiments with these same realistic clocks (Doan & Vigo, 2016). Alternatively, observers can also choose to engage in semi-FR sorting, which is similar to FR sorting but does not result in a category with as much pairwise similarity between members. There are two possible ways per trial to engage in semi-FR behavior and each way creates an instance of structure type III of the 3_2_[4] family.

The third structure type (3_2_[3] – III) does not involve presenting three clocks such that a 1D nor an XOR rule is useful. As an example, we may present the following three clocks: {square/black/few 🡪 square/white/many 🡪 circular/black/many}. Among the three types discussed here, categorization performance with or without feedback is markedly worse for this structure type (Feldman, 2000; Nosofsky et al., 1994; Shepard et al., 1961; Vigo, 2013). However, Doan and Vigo (2016) reveal that observers are consistent in selecting a particular clock among the five (e.g., circular/white/few) when the construction task is parainformative in nature and this selection forms type VI of the 3_2_[4] family. Currently, we will refer to this three-dimensional strategy as C-3D (“Complex 3D”) and we are not aware of any serioinformative unsupervised study that has found consistent selections among participants of this complexity. Interestingly, participants could utilize behavior consistent with FR sorting for this type by selecting the (square/black/many) clock in the example, thus creating structure type IV of the 3_2_[4] family.

Taken together, type I contrasts 1D and FR sorting behavior, type II contrasts XOR and Semi-FR sorting behavior, and type III contrasts C-3D and FR sorting behavior. However, it is also possible for observers to create type V of the 3_2_[4] family for each of these 3_2_[3] structure types. We counterbalanced the presentation of the three levels per participant (e.g., 1/6 of participants received 3_2_[3] – II, followed by 3_2_[3] – I, followed by 3_2_[3] – III) to help control for any practice, fatigue, or carry-over effects.

**Procedure**

Upon giving informed consent, participants were relayed verbally the following information:

Today you are taking part in a study on category construction. You will complete three tasks, each taking place on the computer, where you will be shown images of clock stimuli. For each of the three tasks, you will be shown a series of clocks one-at-a-time on the computer screen. After viewing the series of clocks, you will be presented with a group of five clocks in the middle of the screen. Your task is to select the one clock, out of the five shown, that you believe belongs next in the sequence. Once the main task begins, you will have multiple trials to select clocks and each task should take no longer than 15 minutes to complete.

Now, as you complete each task, we encourage you to be consistent with how you are selecting clocks from the five shown. We will pay you a maximum of $3.00 depending on how consistent you are in selecting the clocks throughout the tasks.

Before starting the main task, you will have a brief training session where you can complete some example trials. The maximum amount of money you can earn will not be affected during the training session. The training session is an opportunity for you to become familiar with the task and to ask me any questions. Once the main task begins, you will have multiple trials to select the clocks you believe belong next in the sequence. Each task should only take about 15 minutes to complete. Are there any questions?

To begin, we are going to give you $2.00. This is yours to keep regardless of how you perform in the task. Again, you can earn up to $3.00 more depending on how consistent you are in selecting the clocks throughout the tasks.

After relaying this information verbally and answering any questions, the researcher gave the participant $2 and guided them to the computer to begin the brief training session. We gave each participant $2 before the experiment started so they were aware (and believed) that they could earn the additional $3 based on their performance in the main task. Lastly, the instructions were again presented on the computer screen for a fixed duration at the start of the training session and each of the main tasks. The purpose of the training session, which was identical in design to the main task, was to familiarize participants with the stimuli and task and involved participants making categorization decisions for two randomly presented instances of each of the three structure types (*N* = 6 trials). The primary dependent variables of interest are the chosen object (realistic clock) and associated response time per trial.

**Results**

We performed a separate 3 (*Task Order*) x 12 (*Block*) mixed ANOVA for each of the three structure types (3_2_[3] – I, II, and III) for both the proportion of selection and response time data (see Figure 3). Each of these multifactor ANOVAs assesses whether there was a difference in 1D, XOR, and C-3D concept completion behavior (*Block*), and whether such a difference in completion behavior was dependent on the order in which participants completed the task (*Task Order*). We restricted our analysis to participants who engaged in these behaviors more than any other behavior, as they were predominantly preferred with their respectively presented 3_2_[3] structure type (1D: 80%; XOR: 68%; C-3D: 54%). The resulting analyses involve concept completion decisions from 53/54, 51/54, and 44/54 of the participants for each of the three concepts, respectively. Finally, we removed response times per block that were more than 2 standard deviations above the mean response time for that block, which results in listwise deletion of these participants in the response time analyses we report below (*N* = 15, 12, and 13 removed for the three structure types, respectively).

Considering 1D completions and creation of structure type 3_2_[4] – I, we found no interaction between the two factors, Greenhouse-Geisser-corrected $F\left( 16.8, 420 \right)=0.66, p=.84, \eta_{p}^{2}=.03$. Additionally, we found no statistical main effect of either *Block* or *Task Order*, least favorable $F\left( 2, 50 \right)=0.93, p=.40, \eta_{p}^{2}=.04$. The plot of these results in the top left of Fig. S1 shows no discernable increase for any of the three groups of participants. Thus, there was no change in 1D concept completions, regardless of the order in which participants completed the task. With respect to response times to make a 1D decision, we found no interaction between the two factors, Greenhouse-Geisser-corrected $F\left( 14.34, 250.95 \right)=1.21, p=.27, \eta_{p}^{2}=.07$. However, we did find a statistical main effect of *Block*, Greenhouse-Geisser-corrected $F\left( 7.17, 250.95 \right)=2.45, p=.018,\eta_{p}^{2}=.07$. Together with the pattern of results in the top right pane of Fig. S1, it appears there was a reduction in response times to engage in 1D completions as the task progressed.


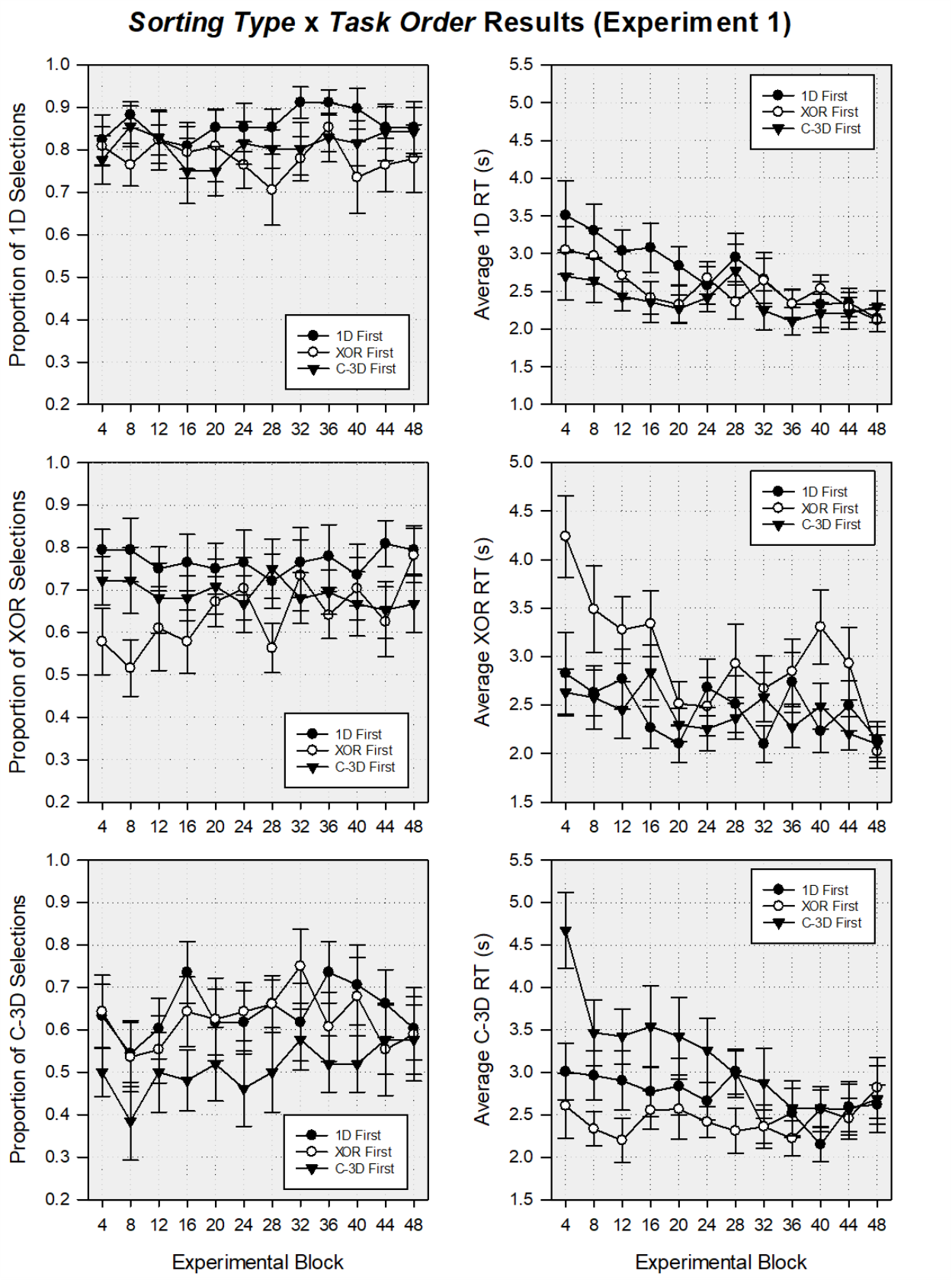


**S1 Figure 1.** **Proportion of 1D, XOR, and C3D concept completions (left column) and associated response times for each trial (right column) across the pilot experiment.** The top, middle, and bottom plots depict performance for each of the three sorting strategies separated by which task was experienced first by participants. Standard error bars are provided in each plot.

Considering XOR concept completions and creation of structure type 3_2_[4] – II, we found no interaction between the two factors, Greenhouse-Geisser-corrected $F\left( 16.45, 394.7 \right)=1.16, p=.30, \eta_{p}^{2}=.05$. Additionally, we found no statistical main effect of either *Block* or *Task Order*, least favorable $F\left( 2, 48 \right)=1.76, p=.18, \eta_{p}^{2}=.07$. Regarding response times, we found a statistical interaction between the two factors, Greenhouse-Geisser-corrected $F\left( 14.54, 261.74 \right)=2.82, p=.005, \eta_{p}^{2}=.11$. Investigating the simple main effects, it appears this interaction is due to a statistical reduction in response times for participants who completed the XOR structure first compared to the participants who did not, $F\left( 11, 110 \right)=3.81, p<.001, \eta_{p}^{2}=.28.$ However, further analysis of the simple main effects reveals that this difference in response times across the three task orders may only be attributable to the set of four decisions made first in the XOR task, $F\left( 2, 36 \right)=9.36,p<.001, \omega^{2}=.30$.

Despite these null results, the plot of these results in the middle left pane of Fig. 3 seems to show an increase in XOR completions for participants who engaged in this task first and the middle right pane of Fig. S1 seems to show a decrease in response times for these same participants. A post-hoc linear regression analysis for these 16 participants revealed a statistical increase in XOR concept completions, $F\left( 1, 10 \right)=8.53, p=.015,{b=.015, 95\% CI [.004, .03], R}^{2}=.46$, and a statistical decrease in response times, $F\left( 1, 10 \right)=8.25, p=.017,{b=-.11, [-.19, -.02], R}^{2}=.45$. These results, with only ~1/3 of our participants, suggest that learning of this two-dimensional relation occurred. Also supporting this interpretation is the slightly elevated XOR concept completions for participants who did not complete this task first (e.g., influenced by carry-over/practice effects).

Finally, considering C-3D concept completions and creation of structure type 3_2_[4] – VI, we found no interaction between the two factors, $F\left( 22, 451 \right)=0.65, p=.89, \eta_{p}^{2}=.03$. Additionally, we found no statistical main effect of either *Block* or *Task Order*, least favorable $F\left( 2, 41 \right)=1.48, p=.24, \omega^{2}=.01$. With respect to response times, we found a statistical interaction between the two factors, $F\left( 22, 308 \right)=2.28, p=.001, \eta_{p}^{2}=.14$. Like the XOR result, simple main effects revealed a statistical reduction in response times for participants who completed the C-3D structure first compared to the participants who did not, $F\left( 11, 66 \right)=3.79, p<.001, \eta_{p}^{2}=.39$. Like the XOR result, further analysis of the simple main effects reveals that this difference in response times may only be attributable to the set of four decisions made first in the C-3D task, $F\left( 2, 28 \right)=10.52,p<.001, \omega^{2}=.38$.

Again, despite these null results, the plot of these results in the bottom left pane of Fig. S1 does seem show an increase in C-3D completions for participants who engaged in this task first and the bottom right pane of Fig. S1 seems to show a decrease in response times for these same participants. A post-hoc linear regression analysis for these 13 participants revealed a statistical increase in C-3D concept completions as the task progressed, $F\left( 1, 10 \right)=10.64, p=.009,{b=.011, 95\% CI [.003, .018], R}^{2}=.52$, and a statistical decrease in response times, $F\left( 1, 10 \right)=36.85, p<.001,{b=-.15, 95\% CI [-.20, -.09], R}^{2}=.79$. These results, with only ~1/3 of our participants, suggest that learning of this three-dimensional relation occurred. Also supporting this interpretation is the slightly elevated C-3D concept completions for participants who did not complete this task first (e.g., influenced by carry-over/practice effects).

**Creating 3_2_[4] Structure Types**

In addition to assessing the presence of multidimensional unsupervised learning, Figure S2 displays the differential creation of the 3_2_[4] structure types across the each of the participants. Importantly, we sorted participants based on their 1D consistency level (i.e., creating 3_2_[4] – I), with those achieving highest rates of consistency being represented as the leftmost bars for the plots in the first column. This order was preserved for the plots in the second and third columns, permitting an analysis of how being consistent with 1D concept completions relates to being consistent with XOR (3_2_[4] – II) and C-3D (3_2_[4] – VI) concept completions.

As shown in Fig. S2, the easiest to learn 3_2_[4] structure type (type I) was created most often (3_2_[3] – I modification), followed by type II (3_2_[3] – II modification), and lastly by type VI (3_2_[3] – III modification). Beyond these consistency results, participants who were more consistent creating type I were also more consistent creating types II and VI, least favorable $r_{s}\left( 52 \right)=.59, p<.001, 95\% CI [.38, .74]$. Additionally, participants who were more consistent creating type II were also more consistent creating type VI, $r_{s}\left( 52 \right)=.78, p<.001, [.65, .87]$. We report Spearman rho analyses here as none of the variables were normally distributed and the visual plots of the relationships between two of the comparisons appeared logarithmic (e.g., 1D *vs.* XOR, 1D *vs.* C-3D).


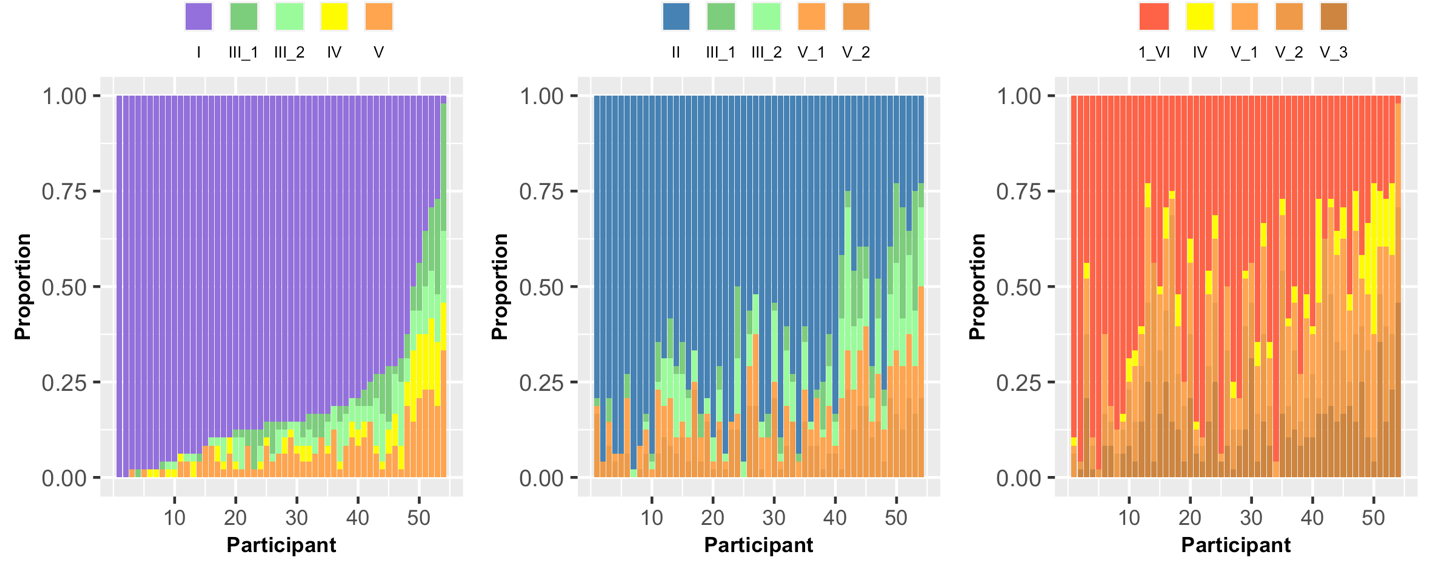


**S1 Figure 2.** **Individual-level creation of the 3_2_[4] structure types across the three structure types (3_2_[3] – I, 3_2_[3] – II, and 3_2_[3] – III) tested in the Pilot study.**

The participants are ordered, left to right, from the most to least consistent in creating 3_2_[4] – I, the easiest to learn of the six structure types. The order of participants for the second and third columns maintains this ordering of consistency. Note that 1D, XOR, and C-3D sorting are represented across the three plots as types I, II, and VI, respectively.

**Discussion**

We observed substantial concept completion aligning with a one-dimensional rule (80% of choices) and a two-dimensional exclusive-or rule (68% of choices) when using such a rule was perfectly applicable. We also observed substantial three-dimensional concept completion associated with the most difficult to learn 3_2_[4] structure – Type VI (54% of choices; “C-3D”). Importantly, all three of these results mirror and extend on much prior research on unsupervised categorization behavior (Ashby et al., 1999; Doan and Vigo, 2016; Love, 2002). More generally, however, this conceptual replication further reveals complex sorting behavior and does so in relation to the often investigated conceptually simple one-dimensional or similarity-based family-resemblance behavior.

However, did participants in the current tasks learn to use the 1D, XOR, or C-3D strategies when completing these concepts? In other words, were these strategies merely preferences from the outset of the experiment? Our results indicate that, at the minimum, perceptual learning occurred for each strategy. This is supported by the significant decrease in response times coupled with the generally unchanging proportion of consistent object selections. But did learning of the relationships between objects occur? Technically, yes, if extracting relational information among the members of a category establishes such preferences in the first place. This follows because with more complex categorical stimuli, observers may reasonably become more sensitive to the underlying relational structure that exists among the members across time. Such an increase in sensitivity would result in both a reduction in response times to categorize and an increase in object selections consistent with the optimal categorization strategy. The current categorical stimuli are relatively simple to learn in related categorization tasks where the categorical information is presented simultaneously (Feldman, 2000; Vigo, 2013); thus, the sensitivity to the relational structure was likely high from the start of the task with each of these three Boolean structures.

Although the majority of 1D, XOR, and C-3D results indicate a lack of change in categorization behavior, we did observe significant increases in XOR and C-3D concept completions for participants who first made their decisions for these particular structures (3_2_[3] – II and III, respectively). This learning cannot be explained via carryover effects that were likely influential after participants experienced a different structure in the first or second task. Instead, these results imply that observers became more sensitive to detecting the structure of the XOR and C-3D relations.
