# Supplementary material for: Constructing concepts without feedback: An empirical investigation of how relational information affects multidimensional concept completion behavior in an unsupervised task
Source: PLoS One. 2025 Aug 7;20(8):e0328368. doi: 10.1371/journal.pone.0328368 (PMC12331049; doi:10.1371/journal.pone.0328368)
Supplement: S2 Appendix — (DOCX) [file pone.0328368.s002.docx]

**S2 Appendix**

**Brief synopsis of the law of invariance and RIT measure**

The law of invariance with λ-equilibrium is shown below. It states that the degree of subjective complexity *ψ* (or degree of learning difficulty) of a categorical stimulus X, where a categorical stimulus is a set of dimensionally defined object stimuli, is directly proportional to the cardinality of the categorical stimulus $\left| X \right|$ and inversely proportional to the exponent of its degree of categorical invariance squared. *D* stands for the number of categories of the category structure and *D*_0_ is the baseline number of dimensions 2. $\lambda$ is the structural equilibrium index which stands for the proportion of dimensions associated with zero invariants. For a detailed explanation of the law, please see Vigo (2013, 2015) and Vigo, Doan, and Zhao (2022).

(A1)

**Representational Information Measure**

In Representational Information Theory (Vigo, 2011) and Generalized Representational Information Theory (Vigo, 2013a, 2015), the amount and quality of subjective information $\hbar_{s}$ conveyed by a subset R of a well-defined category $F$ is defined as the percentage change in the structural complexity of $F$ whenever R is subtracted from $F$. This is expressed by the following equation:

(A2) $\hbar_{s}\left( R | F \right)= \frac{\psi\left( G \right)- \psi\left( F \right)}{\psi\left( F \right)}= \frac{\frac{\left| G \right|{e^{-}}^{{\frac{D_{0}}{D}\Phi}^{2}(G)}}{\lambda}-\frac{\left| F \right|{e^{-}}^{{\frac{D_{0}}{D}\Phi}^{2}(F)}}{\lambda}}{\frac{\left| F \right|{e^{-}}^{{\frac{D_{0}}{D}\Phi}^{2}(F)}}{\lambda}}$

In the equation above, $R\subseteq F$ or $R\in℘(F)$, $G=F-R,$ $\left| G \right|$ and $\left| F \right|$ are the number of elements in $G$ and $F$ respectively, and $\Phi$ is the degree of categorical invariance of a set of well-defined objects (see Vigo, 2009 for a detailed discussion of structural complexity and categorical invariance). As may be seen in the equation above, the degree of complexity $\psi$ of a well-defined category of objects is defined as the product between the number of objects in the set $\left| F \right|$ and the negative exponent of its degree of categorical invariance $\Phi$ (Vigo, 2013, 2015), where $\frac{D_{0}}{D}$ is a discriminability index for structures in which $D$ is the number of dimensions over which the category is defined and $D_{0}$= 2 (the minimum non-trivial number of dimensions that define a category relationally). Equation A2 may be also expressed in additive terms as follows:

(A3)

In the above, and $k=\frac{D_{0}}{D}$ in the variant that does not contain free parameters. Please, note that in Vigo (2015) there is a typographical error with respect to this equation on page 173 where the prime symbol (i.e., single quote or apostrophe symbol) does not show.
