# Supplementary material for: Constructing concepts without feedback: An empirical investigation of how relational information affects multidimensional concept completion behavior in an unsupervised task
Source: PLoS One. 2025 Aug 7;20(8):e0328368. doi: 10.1371/journal.pone.0328368 (PMC12331049; doi:10.1371/journal.pone.0328368)
Supplement: S3 Appendix — (DOCX) [file pone.0328368.s003.docx]

**S3 Appendix**

**Analysis of participant verbal responses during experiment debriefing**

Accompanying the concept completion task results is the verbal description analysis for each of the tested relations (XOR, C-3D). Each participant was asked to describe how they went about completing the concept completion task after they had completed the two tasks. We coded responses for each participant into several categories, such as “Described memorizing the objects” or “Described the stimulus dimensions”, and assessed the proportion of agreement among three raters for these verbal assignments (see Tables 1 and 2 for the categories). These qualitative data are much more varied across participants than the learning results presented in the main text, but we were able to extract the following patterns in the responses.

**Exclusive-or Verbal Analysis**

Regarding this two-dimensional relation, participants generally reported noticing the dimensions of variation and their feature differences (65%), followed by completing a “pattern” or “matching” process (31%). Multiple participants also reported pairing objects together (e.g., 1^st^/2^nd^ and 3^rd^/4^th^) or looking to complete a “pattern”. When pressed further to describe what they meant by “pattern”, many participants described an example sequence and how they would modify it. Some of these participants expressed all three dimensions and values, but there was minimal verbalization of a complete/accurate exclusive-or rule or relation. In addition, very few participants verbalized any statement consistent with a heuristic of looking to add objects that had the least occurring dimensional value across all three dimensions (“balancing features”; 6%). A comprehensive breakdown of interrater agreement for all the considered categories is provided in Table 1.

**S3 Table 1**. **Exclusive-or Verbal Description Categories and Analysis Among Three Raters (N = 48 participants).**

| **Verbal Description Categories** | | | | | | | | | |
| --- | --- | --- | --- | --- | --- | --- | --- | --- | --- |
| 1. Described memorizing the objects (Used term “memorized”) | | | | | | | | | |
| 1. Described the stimulus dimensions and/or their different values | | | | | | | | | |
| 1. Described an example of the XOR relation with the stimulus objects | | | | | | | | | |
| 1. Described completing a “pattern” or a “matching” process | | | | | | | | | |
| 1. Described counting features (dimensional values), selecting the ones that did not occur the most in the series (May have used term “balancing”) | | | | | | | | | |
| 1. Described pairing objects together (2 together, grouping objects together) | | | | | | | | | |
| 1. Other (Seems to be a systematic strategy) | | | | | | | | | |
| 1. Other (Not enough information) | | | | | | | | | |
|  | **Verbal Description Category** | | | | | | | | |
| **Agreement b/w Raters** | **1** | **2** | **3** | **4** | **5** | **6** | **7** | **8** | $\boldsymbol{M}$ |
| 0^a^ | .75 | .35 | .90 | .69 | .94 | .73 | .54 | .69 | **.70** |
| 1 | .15 | .15 | .06 | .06 | .04 | .04 | .25 | .17 | **.12** |
| 2 | .04 | .19 | .04 | .08 | .02 | .19 | .17 | .04 | **.10** |
| 3 | .06 | .31 | .00 | .17 | .00 | .04 | .04 | .10 | **.09** |
| 0 + 3^b^ | **.81** | **.67** | **.90** | **.85** | **.94** | **.77** | **.58** | **.79** | **.79** |

*Note*. There were three raters (Dr. Doan and two undergraduate research assistants) and a value of “3” for agreement indicates all three raters selected that verbal description category.

^a^The value of “0” for agreement does not indicate a lack of agreement across all 3 raters; rather, the value indicates that all three raters found that verbal description to be absent. Thus, it is a form of maximum agreement (for the absence of that verbal description category).

^b^This value per column represents the proportion of agreement across all three raters for identifying the absence of a category (0) and the presence of a category (3).

**Complex-3D Verbal Analysis**

Similar to the exclusive-or relation, for this three-dimensional relation, participants generally reported noticing the dimensions of variation and their feature differences (57%), followed by completing a “pattern” or “matching” process (31%). Multiple participants also reported pairing objects together (e.g., 1^st^/2^nd^ and 3^rd^/4^th^) or looking to complete a “pattern”. When pressed further to describe what they meant by “pattern”, many participants described an example sequence and how they would modify it. In addition, very few participants verbalized any statement consistent with a heuristic of looking to add objects that had the least occurring dimensional value across all three dimensions (“balancing features”; 6%). A comprehensive breakdown of interrater agreement for all the considered categories is provided in Table 2.

**S3 Table 2. Complex 3-D Verbal Description Categories and Analysis Among Three Raters (N = 47 participants.**

| **Verbal Description Categories** | | | | | | | | |  |
| --- | --- | --- | --- | --- | --- | --- | --- | --- | --- |
| 1. Described memorizing the objects (Used term “memorized”) | | | | | | | | |  |
| 1. Described the stimulus dimensions and/or their different values | | | | | | | | |  |
| 1. Described completing a “pattern” or a “matching” process | | | | | | | | |  |
| 1. Described counting features (dimensional values), selecting the ones that did not occur the most in the series (May have used term “balancing”) | | | | | | | | |  |
| 1. Described pairing objects together (2 together, grouping objects together) | | | | | | | | |  |
| 1. Other (Seems to be a systematic strategy) | | | | | | | | |  |
| 1. Other (Not enough information) | | | | | | | | |  |
|  | **Verbal Description Category** | | | | | | | |  |
| **Agreement b/w Raters** | **1** | **2** | **3** | **4** | **5** | **6** | **7** | $\boldsymbol{M}$ | |
| 0^a^ | .79 | .43 | .62 | .94 | .85 | .57 | .89 | **.73** | |
| 1 | .02 | .11 | .02 | .06 | .04 | .19 | .04 | **.07** | |
| 2 | .09 | .09 | .15 | .00 | .04 | .11 | .06 | **.08** | |
| 3 | .11 | .38 | .21 | .00 | .06 | .13 | .00 | **.13** | |
| 0 + 3^b^ | **.89** | **.81** | **.83** | **.94** | **.92** | **.70** | **.89** | **.85** | |

*Note*. There were three raters (Dr. Doan and two undergraduate research assistants) and a value of “3” for agreement indicates all three raters selected that verbal description category.

^a^The value of “0” for agreement does not indicate a lack of agreement across all 3 raters; rather, the value indicates that all three raters found that verbal description to be absent. Thus, it is a form of maximum agreement (for the absence of that verbal description category).

^b^This value per column represents the proportion of agreement across all three raters for identifying the absence of a category (0) and the presence of a category (3).
