# Supplementary material for: Constructing concepts without feedback: An empirical investigation of how relational information affects multidimensional concept completion behavior in an unsupervised task
Source: PLoS One. 2025 Aug 7;20(8):e0328368. doi: 10.1371/journal.pone.0328368 (PMC12331049; doi:10.1371/journal.pone.0328368)
Supplement: S4 Appendix — (DOCX) [file pone.0328368.s004.docx]

**S4 Appendix**

**Full results of all regression analyses (coefficients, standard error, p-values, confidence intervals, goodness-of-fit)**

**Regression results for XOR and C-3D concept completions and associated response times per trial**

The results reported in this appendix accompany the main analyses reported in the manuscript, as they reflect parameter estimates and additional statistical measures. Tables 1 and 2 below display statistics of the simple linear and logarithmic regression analyses conducted for the XOR concept completion task, with Table 1 referencing statistics associated with proportion of XOR completions and Table 2 referencing statistics associated with response times across trials for the XOR completion tasks. Tables 3 and 4 below display statistics of the simple linear and logarithmic regression analyses conducted for the C-3D concept completion task, with Table 3 referencing statistics associated with proportion of C-3D completions and Table 4 referencing statistics associated with response times across trials for the C-3D completion tasks.

**S4 Table 1. Inferential statistics of simple linear/logarithmic regression analyses for proportion of XOR concept completions.**

| **Task** | **Stimulus** | ***b_0_***  **(SE)** | ***95% CI b_0_*** | ***b_1_***  **(SE)** | ***95% CI b_1_*** | ***F*** | ***p*** | $\boldsymbol{R}^{\boldsymbol{2}}$  **(RMSE)** |
| --- | --- | --- | --- | --- | --- | --- | --- | --- |
| First  (12 Blocks) | Clocks | .58  (.03) | [.52, .65] | .008  (.004) | [-.0035, .017] | 4.58 | .058* | .31  (.047) |
|  | T-shirts | .52  (.02) | [.47, .57] | .016  (.003) | [.009, .022] | 30.05 | <.001 | .75  (.034) |
|  | Combined | .55  (.014) | [.52, .58] | .012  (.002) | [.008, .016] | 37.12 | <.001 | .79  (.023) |
| Second  (12 Blocks) | Clocks | .64  (.032) | [.57, .72] | .005  (.004) | [-.005, .015] | 1.44 | .26 | .13  (.053) |
|  | T-shirts | .72  (.019) | [.68, .77] | -.0004  (.003) | [-.006, .005] | 0.02 | .88 | .002  (.031) |
|  | Combined | .69  (.017) | [.65, .72] | .002  (.002) | [-.003, .007] | 1.08 | .32 | .10  (.027) |
| Both  (24 Blocks) | Combined  (Linear) | .59  (.013) | [.56, .61] | .006  (.0009) | [.004, .008] | 49.23 | <.001 | .69  (.03) |
|  | Combined^  (Logarithmic) | .53  (.014) | [.50, .56] | .058  (.006) | [.046, .07] | 107.7 | <.001 | .83  (.022) |

*Note*. The tasks were counterbalanced such that participants were randomly assigned to complete either 48 trials (12 blocks of 4 trials) for the clock stimuli or the t-shirt stimuli before completing 48 trials for the other stimulus type. Each of the scatterplots displayed a linear relationship between the IV (# of Blocks) and proportion of XOR completions, except for when both tasks and stimuli were combined across participants (logarithmic; see ^). Each distribution of proportion of XOR concept completions satisfied the normality assumption as determined by a Shapiro-wilk test (all $p^{'}s> .05$).

**p* < .05 assuming one-tailed alternative hypothesis

**S4 Table 2. Inferential statistics of simple linear/logarithmic regression analyses for response times across trials of the XOR concept completion tasks.**

| **Task** | **Stimulus** | ***b_0_***  **(SE)** | ***95% CI b_0_*** | ***b_1_***  **(SE)** | ***95% CI b_1_*** | ***F*** | ***p*** | $\boldsymbol{R}^{\boldsymbol{2}}$  **(RMSE)** |
| --- | --- | --- | --- | --- | --- | --- | --- | --- |
| First  (12 Blocks) | Clocks^  (Logarithmic) | 5.4  (.32) | [4.69, 6.11] | -1.02  (.18) | [-1.41, -.63] | 33.89 | <.001 | .77  (.44) |
|  | T-shirts | 4.78  (.23) | [4.27, 5.3] | -.15  (.031) | [-.22, -.08] | 22.34 | <.001 | .69  (.38) |
|  | Combined^  (Logarithmic) | 5.19  (.18) | [4.79, 5.6] | -.86  (.10) | [-1.09, -.64] | 73.66 | <.001 | .88  (.25) |
| Second  (12 Blocks) | Clocks | 3.6  (.17) | [3.22, 3.97] | -.067  (.023) | [-.12, -.02] | 8.67 | .015 | .46  (.27) |
|  | T-shirts | 3.06  (.11) | [2.81, 3.31] | -.034  (.015) | [-.068,-.0006] | 5.15 | .047 | .34  (.18) |
|  | Combined | 3.32  (.057) | [3.19, 3.45] | -.05  (.008) | [-.067, -.032] | 40.62 | <.001 | .80  (.093) |
| Both  (24 Blocks) | Combined  (Linear) | 4.29  (.16) | [3.97, 4.62] | -.073  (.011) | [-.096, -.051] | 44.79 | <.001 | .67  (.37) |
|  | Combined^  (Logarithmic) | 5.02  (.13) | [4.76, 5.29] | -.72  (.053) | [-.83, -.61] | 188.2 | <.001 | .90  (.21) |

*Note*. The tasks were counterbalanced such that participants were randomly assigned to complete either 48 trials (12 blocks of 4 trials) for the clock stimuli or the t-shirt stimuli before completing 48 trials for the other stimulus type. Most of the scatterplots displayed a linear relationship between the IV (# of Blocks) and the time to respond on each trial. There appeared to be a logarithmic relationship for Ps who made decisions for the clock stimuli first, for the data set that combined the stimulus types for the first task, and for the combined data set across both tasks and stimulus types (see ^). Most distribution of times to respond on each trial satisfied the normality assumption as determined by a Shapiro-wilk test (all $p^{'}s> .05$), except for Ps who made decisions for the clock stimuli first ($p<.001$) and for the combined response times for the first task ($p=.007$).

**p* < .05 assuming one-tailed alternative hypothesis

**S4 Table 3. Inferential statistics of simple linear regression analyses for proportion of C-3D concept completions.**

| **Task** | **Stimulus** | ***b_0_***  **(SE)** | ***95% CI b_0_*** | ***b_1_***  **(SE)** | ***95% CI b_1_*** | ***F*** | ***p*** | $\boldsymbol{R}^{\boldsymbol{2}}$  **(RMSE)** |
| --- | --- | --- | --- | --- | --- | --- | --- | --- |
| First  (12 Blocks) | Clocks | .39  (.04) | [.30, .48] | .018  (.005) | [.006, .03] | 10.71 | .008 | .52  (.065) |
|  | T-shirts | .46  (.03) | [.40, .53] | .011  (.004) | [.002, .02] | 8.06 | .018 | .45  (.048) |
|  | Combined | .43  (.026) | [.37, .49] | .015  (.004) | [.007, .023] | 16.4 | .002 | .62  (.043) |
| Second  (12 Blocks) | Clocks | .58  (.035) | [.50, .65] | .011  (.005) | [.0007, .022] | 5.66 | .039 | .36  (.056) |
|  | T-shirts | .66  (.028) | [.59, .72] | .002  (.004) | [-.007, .01] | 0.25 | .63 | .02  (.046) |
|  | Combined | .62  (.026) | [.56, .67] | .007  (.003) | [-.001, .014] | 3.65 | .085* | .27  (.042) |
| Both  (24 Blocks) | Combined  (Linear) | .45  (.018) | [.41, .49] | .011  (.001) | [.009, .014] | 79.21 | <.001 | .78  (.043) |

*Note*. The tasks were counterbalanced such that participants were randomly assigned to complete either 48 trials (12 blocks of 4 trials) for the clock stimuli or the t-shirt stimuli before completing 48 trials for the other stimulus type. Each of the scatterplots displayed a linear relationship between the IV (# of Blocks) and proportion of XOR completions. Each distribution of proportion of C-3D concept completions satisfied the normality assumption as determined by a Shapiro-wilk test (all $p^{'}s> .05$).

**p* < .05 assuming one-tailed alternative hypothesis

**S4 Table 4. Inferential statistics of simple linear/logarithmic regression analyses for response times across trials of the C-3D concept completion tasks.**

| **Task** | **Stimulus** | ***b_0_***  **(SE)** | ***95% CI b_0_*** | ***b_1_***  **(SE)** | ***95% CI b_1_*** | ***F*** | ***p*** | $\boldsymbol{R}^{\boldsymbol{2}}$  **(RMSE)** |
| --- | --- | --- | --- | --- | --- | --- | --- | --- |
| First  (12 Blocks) | Clocks | 4.93  (.28) | [4.29, 5.56] | -.18  (.039) | [-.27, -.10] | 22.31 | <.001 | .69  (.46) |
|  | T-shirts | 5.02  (.24) | [4.48, 5.56] | -.19  (.033) | [-.27, -.12] | 34.55 | <.001 | .78  (.39) |
|  | Combined | 4.97  (.24) | [4.44, 5.5] | -.19  (.032) | [-.26, -.12] | 33.87 | <.001 | .77  (.39) |
| Second  (12 Blocks) | Clocks | 3.17  (.12) | [2.89, 3.44] | -.048  (.017) | [-.085, -.011] | 8.3 | .016 | .45  (.20) |
|  | T-shirts | 3.25  (.15) | [2.93, 3.58] | -.052  (.02) | [-.096, -.008] | 6.87 | .026 | .41  (.24) |
|  | Combined | 3.21  (.12) | [2.94, 3.49] | -.05  (.017) | [-.087, -.013] | 8.95 | .014 | .47  (.20) |
| Both  (24 Blocks) | Combined  (Linear) | 4.37  (.17) | [4.01, 4.73] | -.084  (.012) | [-.11, -.059] | 48.22 | <.001 | .69  (.41) |
|  | Combined^  (Logarithmic) | 5.17  (.15) | [4.85, 5.48] | -.81  (.063) | [-.94, -.68] | 164.2 | <.001 | .88  (.25) |

*Note*. The tasks were counterbalanced such that participants were randomly assigned to complete either 48 trials (12 blocks of 4 trials) for the clock stimuli or the t-shirt stimuli before completing 48 trials for the other stimulus type. Each of the scatterplots displayed a linear relationship between the IV (# of Blocks) and the time to respond on each trial, except for the combined data set across both tasks and stimulus types (logarithmic; see ^). Each distribution of proportion of C-3D concept completions satisfied the normality assumption as determined by a Shapiro-wilk test (all $p^{'}s> .05$), except for the combined data set across both tasks and stimulus types ($p=.002$).

**p* < .05 assuming one-tailed alternative hypothesis

**Regression results for GRIT model fits to the 1D, XOR, and C-3D concept completion data**

Table 5 below displays the statistics of the simple linear regression analyses conducted whereby the GRIT-NPE is used to predict the proportion of object selections for each of the concept completion task conditions (1D, XOR, and C-3D). The statistics displayed in Table 5 accompany the results reported in Table 1 in the Discussion section of the manuscript.

**S4 Table 5. Inferential statistics of simple linear regression analyses of the GRIT-NPE as a predictor of behavior in the 1D, XOR, and C-3D concept completion tasks.**

| **Boolean**  **Structure** | **Expt** | ***b_0_***  **(SE)** | ***95% CI b_0_*** | ***b_1_***  **(SE)** | ***95% CI b_1_*** | ***F*** | ***p*** | $\boldsymbol{R}^{\boldsymbol{2}}$  **(RMSE)** |
| --- | --- | --- | --- | --- | --- | --- | --- | --- |
| 3_2_[3] – I  (**1D**) | Pilot | .39  (.007) | [.37, .41] | .72  (.014) | [.68, .76] | 2778.97 | <.001 | .99  (.013) |
| 3_2_[3] – II  (**XOR**) | Pilot | .57  (.031) | [.47, .67] | 1.17  (.083) | [.90, 1.43] | 198.65 | <.001 | .99  (.038) |
|  | Main | .55  (.03) | [.46, .65] | 1.12  (.08) | [.87, 1.38] | 196.63 | <.001 | .99  (.037) |
| 3_2_[3] – III  (**C-3D**) | Pilot | 1.22  (.44) | [-.17, 2.62] | 3.33  (1.42) | [-1.18, 7.85] | 5.52 | .10 | .65  (.13) |
|  | Main | 1.31  (.52) | [-.36, 2.98] | 3.64  (1.7) | [-1.76, 9.04] | 4.6 | .12 | .61  (.16) |

*Note*. There are only 5 pairs of observations per simple linear regression analysis.
